# Estimating genomic relationships of metafounders across and within breeds using maximum likelihood, pseudo-expectation–maximization maximum likelihood and increase of relationships

**DOI:** 10.1186/s12711-024-00892-9

**Published:** 2024-05-02

**Authors:** Andres Legarra, Matias Bermann, Quanshun Mei, Ole F. Christensen

**Affiliations:** 1 CDCB, 4201 Northview Drive, Bowie, MD 20716 USA; 2 Animal and Dairy Science, University of Georgia, 425 River Rd, Athens, GA 30602 USA; 3 Department of Biostatistics, Boston University School of Public Health, Boston, MA 02118 USA; 4 Center for Quantitative Genetics and Genomics, Aarhus University, C. F. Møllers Allé 3, bld. 1130, 8000 Aarhus C, Denmark

## Abstract

**Background:**

The theory of “metafounders” proposes a unified framework for relationships across base populations within breeds (e.g. unknown parent groups), and base populations across breeds (crosses) together with a sensible compatibility with genomic relationships. Considering metafounders might be advantageous in pedigree best linear unbiased prediction (BLUP) or single-step genomic BLUP. Existing methods to estimate relationships across metafounders ${\varvec{\Gamma}}$ are not well adapted to highly unbalanced data, genotyped individuals far from base populations, or many unknown parent groups (within breed per year of birth).

**Methods:**

We derive likelihood methods to estimate ${\varvec{\Gamma}}$. For a single metafounder, summary statistics of pedigree and genomic relationships allow deriving a cubic equation with the real root being the maximum likelihood (ML) estimate of ${\varvec{\Gamma}}$. This equation is tested with Lacaune sheep data. For several metafounders, we split the first derivative of the complete likelihood in a term related to ${\varvec{\Gamma}}$, and a second term related to Mendelian sampling variances. Approximating the first derivative by its first term results in a pseudo-EM algorithm that iteratively updates the estimate of ${\varvec{\Gamma}}$ by the corresponding block of the **H**-matrix. The method extends to complex situations with groups defined by year of birth, modelling the increase of ${\varvec{\Gamma}}$ using estimates of the rate of increase of inbreeding ($\Delta F$), resulting in an expanded ${\varvec{\Gamma}}$ and in a pseudo-EM+$\Delta F$ algorithm. We compare these methods with the generalized least squares (GLS) method using simulated data: complex crosses of two breeds in equal or unsymmetrical proportions; and in two breeds, with 10 groups per year of birth within breed. We simulate genotyping in all generations or in the last ones.

**Results:**

For a single metafounder, the ML estimates of the Lacaune data corresponded to the maximum. For simulated data, when genotypes were spread across all generations, both GLS and pseudo-EM(+$\Delta F$) methods were accurate. With genotypes only available in the most recent generations, the GLS method was biased, whereas the pseudo-EM(+$\Delta F$) approach yielded more accurate and unbiased estimates.

**Conclusions:**

We derived ML, pseudo-EM and pseudo-EM+$\Delta F$ methods to estimate ${\varvec{\Gamma}}$ in many realistic settings. Estimates are accurate in real and simulated data and have a low computational cost.

## Background

The theory of “metafounders” (abbreviated MF in the following) [1, 2] proposes a unified framework for relationships across base populations within breeds (that are usually modelled using unknown parent groups for different pathways of selection and periods), and base populations across breeds e.g. in crossbred animals (that are also sometimes modelled with unknown parent groups) together with a sensible compatibility with genomic relationships. Relationships across base populations are defined using an “absolute” reference point which is an ideal population with allele frequencies at biallelic markers of 0.5 [3, 4]. These relationships are contained in a matrix called ${\varvec{\Gamma}}$. In essence, matrix ${\varvec{\Gamma}}$ contains average (unobserved) relationships across (unobserved) pools of founder gametes, and these are the so-called metafounders.

It is of interest to use metafounders in predictions that include pedigree, either in pedigree best linear unbiased prediction (BLUP) or (more commonly) in single-step genomic BLUP. The reasons are to obtain both more accurate, less biased, and more robust solutions of metafounders themselves, in particular in the presence of a genetic trend [5, 6], while at the same time ensuring compatibility of pedigree relationships with genomic relationships. This requires an estimate of matrix ${\varvec{\Gamma}}$, which is typically based on genotyped individuals that rarely belong to the base populations of interest.

Legarra et al. [1] suggested a series of methods, which were improved, first, by the discovery that ${\varvec{\Gamma}}$ is actually a function of base allele frequencies [3] and, second, by modelling the increase of relationships within breed [6–9].

Still, there is no consensus and computational efficient methods are lacking. This is true in particular for pedigrees composed of several breeds, possibly with crossings, and with MF defined within and across breeds. For instance, in their study, Kudinov et al. [6] considered in genetic evaluations of four dairy cattle breeds (Holstein, Nordic Red Dairy Cattle, Finncattle and “Other”), each of them, in turn, including 16 to 61 MF. Their method requires that the genotypes are well distributed in time to fit a covariance function across all unknown parent groups. Wicki et al. [10] considered two sub-populations of Lacaune dairy sheep, each with 22 MF; the method uses pedigree inbreeding to model the steady increase in ${\varvec{\Gamma}}$, but requires estimates of ${\varvec{\Gamma}}$ at the earliest generation. All current methods have drawbacks: either they require that MF are within short genetic (time) distances of genotyped individuals [3], or that genotypes are distributed in time to obtain ${\varvec{\Gamma}}$ from a covariance function [6], or methods are adapted to particular cases [10]. Moreover, some methods can provide estimates that are outside of the admissible parametric space (matrix ${\varvec{\Gamma}}$ must be positive semidefinite, and diagonal elements must be within the range from 0 to 2).

The companion paper [11] shows that a definition of ${\varvec{\Gamma}}$ in a quantitative genetics context is such that ${\Gamma }_{i,j}=\frac{2}{k}\left(2{\mathbf{p}}_{i}-\mathbf{1}\right){\left(2{\mathbf{p}}_{j}-\mathbf{1}\right)}^{\prime}$, with ${\mathbf{p}}_{i}$ and ${\mathbf{p}}_{j}$ being the row vectors of allele frequencies of $k$ markers in base populations $i$ and $j$, i.e. ${\varvec{\Gamma}}$ is a “genomic relationship” that is based on “genotypes” of their population, i.e. allele frequencies. Using this result and new developments, here we present: (1) a Maximum Likelihood (ML) estimation for a single MF, (2) an estimation of ${\varvec{\Gamma}}$ for several MF by pseudo-Expectation–Maximization (pseudo-EM) (actually, EM of part of the derivative of the complete log-likelihood), which involves repeated set-ups of part of matrix $\mathbf{H}$-inverse, and (3) in the case of MF structured by year of birth, we couple the pseudo-EM with a heuristic method for within-breed estimation of ${\varvec{\Gamma}}$. We describe the theory and examine the results obtained with a simulated dataset.

## Theory

### Likelihood

The likelihood of given markers is as follows [1, 4]. Let’s define, ${\mathbf{A}}_{\Gamma 22}$, the pedigree relationship matrix of genotyped individuals set up with the MF relationship matrix ${\varvec{\Gamma}}$. Thus, we estimate an unobserved quantity ${\varvec{\Gamma}}$ using a statistical model that involves ${\varvec{\Gamma}}$ as a parameter. We assume Gaussian distributions for convenience. The joint density of the observed genotypes in matrix $\mathbf{Z}=\left[{\mathbf{z}}_{1},...,{\mathbf{z}}_{k}\right]$ with $z$ coded as $\{-{1,0},1\}$, assuming multivariate normality for markers, is, for $k$ markers and given ${\varvec{\Gamma}}$ and pedigree:

$$f\left(\mathbf{M}|{\varvec{\Gamma}}\right)\propto \prod_{j=1}^{k}det{\left(0.5{\mathbf{A}}_{\Gamma 22}\right)}^{-1/2}exp\left(-\mathbf{z}^{\prime}_{j}{\left(0.5{\mathbf{A}}_{\Gamma 22}\right)}^{-1}{\mathbf{z}}_{j}/2\right),$$

where a proportionality constant is ignored. Because the product of exponential terms is the exponential of the sum, and that $\sum_{j}{\mathbf{z}}_{j}^{\prime}{\left(0.5{\mathbf{A}}_{\Gamma 22}\right)}^{-1}{\mathbf{z}}_{j}/2=Tr\left({\left({\mathbf{A}}_{\Gamma 22}\right)}^{-1}\mathbf{Z}{\mathbf{Z}}^{\mathbf{\prime}}\right)$, the likelihood function is:

$$L\left({\varvec{\Gamma}}\right)\propto det{\left(0.5{\mathbf{A}}_{\Gamma 22}\right)}^{-\frac{k}{2}}{exp}\left(-Tr\left({\left({\mathbf{A}}_{\Gamma 22}\right)}^{-1}\mathbf{Z}{\mathbf{Z}}^{\mathbf{\prime}}\right)\right).$$

Taking the logarithm of the likelihood function and introducing the notation $\mathbf{G}=\mathbf{Z}{\mathbf{Z}}^{\mathbf{\prime}}/\left(k/2\right)$, we obtain the log-likelihood function:

$$lo{g}_{L}\left({\varvec{\Gamma}}\right)={\text{constant}}-\left(\frac{k}{2}\right)log\left(det\left({\mathbf{A}}_{\Gamma 22}\right)\right)-\left(\frac{k}{2}\right)Tr\left({\mathbf{A}}_{\Gamma 22}^{-1}\mathbf{G}\right).$$

Note that the likelihood uses $\mathbf{G}$, i.e. a direct crossproduct of marker readings, and the inverse of $\mathbf{G}$ is not needed, so $\mathbf{G}$ does not need to be strictly positive definite. Some options to compute $l\left({\varvec{\Gamma}}\right)$ are presented in Appendix. The constant in the log-likelihood function is invariant to ${\varvec{\Gamma}}$ and is ignored in the following.

Maximization of this likelihood function has proven difficult for the general case. The main difficulty compared to variance component estimation is, first, that it is not possible to factorize ${\mathbf{A}}_{\Gamma 22}$ into a Kronecker product of a parameter-free relationship matrix and ${\varvec{\Gamma}}$, and second, that the elements of ${\varvec{\Gamma}}$ are propagated through the Mendelian sampling variance of the animals. Furthermore, derivative-free and Markov chain Monte Carlo methods proved to be difficult to use with simulated data (not shown). Below, we show an exact solution for a single MF, an EM maximization using part of the derivative of the complete log-likelihood function, and a heuristic extension to within-breed across-time metafounders.

### Maximum likelihood for a single metafounder

The theory for a single MF was presented in [12] and we reintroduce it here for completion and for later discussion. For the case of a single MF, there is an explicit solution that maximizes the log-likelihood function $lo{g}_{L}\left({\varvec{\Gamma}}\right)=-\left(\frac{k}{2}\right)log\left(det\left({\mathbf{A}}_{\Gamma 22}\right)\right)-\left(\frac{k}{2}\right)Tr\left({\mathbf{A}}_{\Gamma 22}^{-1}\mathbf{G}\right)$. Let us call $\gamma$ the (single) scalar value of ${\varvec{\Gamma}}$ for the single MF case. In that case, ${\mathbf{A}}_{\gamma 22}={\mathbf{A}}_{22}\left(1-\frac{\gamma }{2}\right)+{\mathbf{11}}^{\prime}\gamma$, where ${\mathbf{A}}_{22}$ is the matrix of pedigree relationships across genotyped individuals. As detailed in Appendix, we used this to obtain, in short-hand notation, $a=\mathbf{1}^{\prime}{\mathbf{A}}_{22}^{-1}\mathbf{1}$, $b=Tr\left({\mathbf{A}}_{22}^{-1}\mathbf{G}\right)$ and $c=Tr\left({\mathbf{A}}_{22}^{-1}\mathbf{11}^{\prime}{\mathbf{A}}_{22}^{-1}\mathbf{G}\right)=\mathbf{1}^{\prime}{\mathbf{A}}_{22}^{-1}\mathbf{G}{\mathbf{A}}_{22}^{-1}\mathbf{1}$, which are later used in the cubic equation ($n$ being the number of individuals with a genotype):

$${e}_{3}{\gamma }^{3}+{e}_{2}{\gamma }^{2}+{e}_{1}\gamma +{e}_{o}=0,$$

where $${e}_{3}=-n{\left(-1/2+a\right)}^{2}/2$$, $${e}_{2}=n\left(\left(-3/2+a\right)+a-b\left(-1/2+a\right)+c\right)\left(-1/2+a\right)$$, $${e}_{1}=\left(n-1\right)\left(-3/2+2a\right)-\left(-1+2a\right)\left(-3/2+a+b\right)$$ and $${e}_{0}=n-2a-b+2c$$.

The real roots of this equation are the ML estimate of $\gamma$, in our simulated and real examples we have only found one real root. In addition, if the ML estimate of $\gamma$ is outside the parametric space, the estimate is at the boundary. Some methods to compute $b$ and $c$ are presented in Appendix and their estimation is actually easy when matrices $\mathbf{G}$ and ${\mathbf{A}}_{22}^{-1}$ can be explicitly computed.

### Maximum likelihood with multiple metafounders

Here we just sketch what could be done in principle. Using the same log-likelihood, it is conceptually possible to split the likelihood among breeds and pairs of breeds using partial relationship matrices [1, 13]:

$$\begin{aligned} {\mathbf{A}}_{{{\varvec{\Gamma}}}} & = \sum \limits_{b} {\mathbf{A}}^{b} \left( {1 - \frac{{\gamma_{b} }}{2}} \right) + \mathop \sum \limits_{{b,b^{\prime},b^{\prime} > b}} {\mathbf{A}}^{{b,b^{\prime}}} \left( {\frac{{\gamma_{b}^{\prime} + \gamma_{b} }}{8} - \frac{{\gamma_{{b,b^{\prime}}} }}{4}} \right) \\ & \quad + \sum \limits_{b} {\mathbf{C}}^{b} \gamma_{b} + \mathop \sum \limits_{{b,b^{\prime},b^{\prime} > b}} {\mathbf{C}}^{{b,b^{\prime}}} \gamma_{{b,b^{\prime}}} , \\ \end{aligned}$$

with ${\mathbf{A}}^{b}$ being the breed $b$ specific partial relationship matrix, ${\mathbf{A}}^{b,b^{\prime}}$ being the matrix of partial relationships due to segregation across breeds $b$ and $b^{\prime}$ [13], matrix ${\mathbf{C}}^{b}$ having entries ${{\mathbf{C}}}_{i,i^{\prime}}^{b}={f}_{i}^{b}{f}_{i^{\prime}}^{b}$, and matrix ${\mathbf{C}}^{b,{b}^{\prime}}$ having entries ${\mathbf{C}}_{{i,i}^{\prime}}^{b,{b}^{\prime}}={f}_{i}^{b}{f}_{i^{\prime}}^{b^{\prime}}+{f}_{i}^{b^{\prime}}{f}_{i^{\prime}}^{b}$ for ${f}_{i}^{b}$ being the fraction of breed “$b$” origin of individual $i$. From this expression, matrix derivatives with respect to MF parameters can be obtained. However, this has proven to be difficult because the expressions quickly get too complicated.

### Derivatives of the “complete” likelihood

Following expectation–maximization ideas, we consider the derivatives of a “complete” likelihood in which all animals (including MF) are genotyped. The particular block of genomic relationships for MF will be named ${\mathbf{G}}_{MF}$, and indeed by definition ${\mathbf{G}}_{MF}={\varvec{\Gamma}}$ as described in the companion paper [11]. From [11], we know the definition of elements of ${\varvec{\Gamma}}$, ${{\varvec{\Gamma}}}_{b,{b}^{\prime}}=\frac{2}{k}\left(2{{\mathbf{p}}}_{b}-\mathbf{1}\right)\left(2{{\mathbf{p}}}_{{b}^{\prime}}-\mathbf{1}\right)^{\prime}$ for the ${{\mathbf{p}}}_{b}$ and ${{\mathbf{p}}}_{{b}^{\prime}}$ row vectors of allele frequencies, although these allele frequencies are typically unknown (if they are known, estimation is immediate). In other words, it is meaningful to assign genomic relationships to MF. Consider now the form of the “complete” log-likelihood where ${\mathbf{A}}_{\Gamma }$ and $\mathbf{G}$ include all animals and the MF:

$$lo{g}_{L}\left({\varvec{\Gamma}}\right)=-\frac{k}{2}log\left(det\left({\mathbf{A}}_{\varvec{\Gamma} }\right)\right)-\frac{k}{2}Tr\left({\mathbf{A}}_{\varvec{\Gamma} }^{-1}\mathbf{G}\right)=-\frac{k}{2}\left[log\left(det\left({\mathbf{A}}_{\varvec{\Gamma} }\right)\right)+Tr\left({\mathbf{A}}_{\varvec{\Gamma} }^{-1}\mathbf{G}\right)\right].$$

To maximize this “complete” log-likelihood function, we need to derive formulas of the derivatives of $log(det\left({\mathbf{A}}_{\varvec{\Gamma} }\right)$ and $Tr\left({\mathbf{A}}_{\varvec{\Gamma} }^{-1}\mathbf{G}\right)$ with respect to elements in ${\varvec{\Gamma}}$. For convenience, we use $\gamma^ {\prime}s$ to represent each of the several parameters in ${\varvec{\Gamma}}$ in the following.

Consider matrix ${\mathbf{A}}_{{\varvec{\Gamma}}}=\mathbf{T}{\mathbf{D}}_{{\varvec{\Gamma}}}\mathbf{T}^{\prime}$. This matrix includes relationship among individuals, among MF, and among both individuals and MF. Its inverse is ${\left({\mathbf{A}}_{{\varvec{\Gamma}}}\right)}^{-1}=\left({\mathbf{T}}^{-1}\right)^{\prime}{\mathbf{D}}_{{\varvec{\Gamma}}}^{-1}{\mathbf{T}}^{-1}$ with ${\mathbf{T}}^{-1}=\mathbf{I}-\mathbf{S}$ linking individuals to ancestors and MF to themselves; for instance an individual with one parent known and the other a MF has a -0.5 in the (individual, MF) element of $\mathbf{S}$ [14] (where matrix $\mathbf{S}$ was called $\mathbf{P}$). The dependence of $\left({\mathbf{T}}^{-1}\right)^{\prime}{\mathbf{D}}_{{\varvec{\Gamma}}}^{-1}{\mathbf{T}}^{-1}$ on ${\varvec{\Gamma}}$ is only through matrix ${\mathbf{D}}_{{\varvec{\Gamma}}}$.

Matrix ${\mathbf{D}}_{{\varvec{\Gamma}}}$ is a block diagonal matrix, consisting of the ${\varvec{\Gamma}}$ matrix for MF and the usual diagonal matrix with Mendelian sampling terms for non-metafounders; $\mathbf{T}$ and ${\mathbf{T}}^{-1}$ are lower triangular matrices.

After some algebra that is shown in Appendix, we can show that:

$$\frac{\partial {{\text{log}}}_{L}\left({\varvec{\Gamma}}\right)}{\partial \gamma }=-\frac{k}{2}\left[Tr\left[{{\varvec{\Gamma}}}^{-1}\frac{\partial{\varvec{\Gamma}}}{\partial \gamma }\left(\mathbf{I}-{{\varvec{\Gamma}}}^{-1}{\mathbf{G}}_{{\text{MF}}} \right)\right]\right]-\frac{k}{2}\left[\sum_{i}\left[\frac{\partial {D}_{{\Gamma }_{i,i}}}{\partial \gamma }\left(\frac{1}{{D}_{{\Gamma }_{i,i}}}-\frac{{B}_{i,i}}{{D}_{{\Gamma }_{i,i}}^{2}}\right)\right]\right].$$

The structure of $\mathbf{B}$ and ${\mathbf{D}}_{{\varvec{\Gamma}}}$ is:

$${\mathbf{D}}_{{\varvec{\Gamma}}}=\left\{\begin{array}{ll}{\varvec{\Gamma}},& \text{for}\; \text{metafounders}\\ \text{diagonal}\; \text{matrix},& \text{for}\; \text{non-metafounders}\end{array},\right.$$

$$\mathbf{B}={\mathbf{WW}}^{\mathbf{\prime}}=\left\{\begin{array}{ll}{\mathbf{G}}_{MF},& \text{for}\; \text{metafounders}\\ {\mathbf{W}}_{NMF}{\mathbf{W}}_{NMF}^{\prime},& \text{for}\; \text{non-metafounders}\end{array}\right.,$$

where $\mathbf{W}={\mathbf{T}}^{-1}\mathbf{Z}/\sqrt{k/2}$. Note that the block of ${\mathbf{T}}^{-1}$ corresponding to MF is simply an identity matrix, and thus the block of $\mathbf{B}$ corresponding to MF is simply ${\mathbf{G}}_{MF}$.

As for matrix ${\mathbf{G}}_{MF}$, it is the genomic relationships across MF: in order to derive the algorithm, we use the complete likelihood, in which it is assumed that all individuals, including MF, have been genotyped.

The first term in the partial derivative involves ${\mathbf{G}}_{MF}$ and is simple to manipulate. The last term in the partial derivative involves individual terms of Mendelian sampling variances ${D}_{{\Gamma }_{i,i}}$ and it is difficult to compute and derive algebraically in a recognizable form. Instead, we approximate $\frac{\partial lo{g}_{L}\left({\varvec{\Gamma}}\right)}{\partial \gamma }$ by the first term, i.e. as:

$$\begin{array}{c}\frac{\partial lo{g}_{L}\left({\varvec{\Gamma}}\right)}{\partial \gamma }\approx -\frac{k}{2}\left[Tr\left[{{\varvec{\Gamma}}}^{-1}\frac{\partial{\varvec{\Gamma}}}{\partial \gamma }\left(\mathbf{I}-{{\varvec{\Gamma}}}^{-1}{\mathbf{G}}_{MF}\right)\right]\right].\end{array}$$

### Pseudo-EM algorithm

#### E-step

The EM algorithm uses the expectation of the log-likelihood over the distribution of unknown data, conditional to the actual value of the parameters (${\varvec{\Gamma}}$). However, we know from the single step theory that (using the notation $o$ for observed and $n$ for not observed, in fact ${\mathbf{G}}_{o,o}$ is $\mathbf{G}$ actually observed) [15]:

$${E}_{G}\left(\mathbf{G}\right)=E\left(\left[\begin{array}{c}{\mathbf{G}}_{n,n}{\mathbf{G}}_{n,o}\\ {\mathbf{G}}_{o,n}{\mathbf{G}}_{o,o}\end{array}\right]\left|{\mathbf{G}}_{o,o}\right.\right)=\left(\begin{array}{c}{\mathbf{H}}_{n,n}{\mathbf{H}}_{n,o}\\ {\mathbf{H}}_{o,n}{\mathbf{H}}_{o,o}\end{array}\right)=\mathbf{H},$$

and in fact, ${\mathbf{H}}_{o,o}={\mathbf{G}}_{o,o}$ Using this together with $E\left(tr\left(\mathbf{A}\right)\right)=tr\left(E\left(\mathbf{A}\right)\right)$ we get:

$$\begin{aligned} E_{G} \left( {log_{L} \left( {{\varvec{\Gamma}}} \right)} \right) & = E_{G} \left( { - \frac{k}{2}\left[ {log\left( {det\left( {{\mathbf{A}}_{{\Gamma }} } \right)} \right) + Tr\left( {{\mathbf{A}}_{{\Gamma }}^{ - 1} {\mathbf{G}}} \right)} \right]} \right) \\ & = - \frac{k}{2}\left[ {log\left( {det\left( {{\mathbf{A}}_{{\Gamma }} } \right)} \right) + E_{G} \left( {Tr\left( {{\mathbf{A}}_{{\Gamma }}^{ - 1} {\mathbf{G}}} \right)} \right)} \right] \\ & = - \frac{k}{2}\left[ {log\left( {det\left( {{\mathbf{A}}_{{\Gamma }} } \right)} \right) + Tr\left( {{\mathbf{A}}_{{\Gamma }}^{ - 1} E_{G} \left( {\mathbf{G}} \right)} \right)} \right] \\ & = - \frac{k}{2}\left[ {log\left( {det\left( {{\mathbf{A}}_{{\Gamma }} } \right)} \right) + Tr\left( {{\mathbf{A}}_{{\Gamma }}^{ - 1} {\mathbf{H}}} \right)} \right] \\ \end{aligned}$$

This means that, in the following derivations, we can use $\mathbf{H}$ (computed at the actual value of ${\varvec{\Gamma}}$) in the place of $\mathbf{G}$.

#### M-step

The approximate first derivative of the complete log-likelihood shown before is:

$$\begin{array}{c}\begin{array}{c}\frac{\partial lo{g}_{L}\left({\varvec{\Gamma}}\right)}{\partial \gamma }\approx -\frac{k}{2}\left[Tr\left[{{\varvec{\Gamma}}}^{-1}\frac{\partial{\varvec{\Gamma}}}{\partial \gamma }\left(\mathbf{I}-{{\varvec{\Gamma}}}^{-1}{\mathbf{G}}_{MF}\right)\right]\right]\end{array}\end{array},$$

and because the previous E-step uses the conditional expectation of $\mathbf{G}$, i.e. $\mathbf{H}$, this becomes:

$$\begin{array}{c}\frac{\partial lo{g}_{L}\left({\varvec{\Gamma}}\right)}{\partial \gamma }\approx -\frac{k}{2}\left[Tr\left[{{\varvec{\Gamma}}}^{-1}\frac{\partial{\varvec{\Gamma}}}{\partial \gamma }\left(\mathbf{I}-{{\varvec{\Gamma}}}^{-1}{\mathbf{H}}_{MF}\right)\right]\right]\end{array},$$

setting to 0, factorizing $-\frac{{\text{k}}}{2}$ and introducing ${\gamma }_{ij}$ to indicate which of the elements of ${\varvec{\Gamma}}$ we work with, gives:

$$Tr\left[{{\varvec{\Gamma}}}^{-1}\frac{\partial{\varvec{\Gamma}}}{\partial {\gamma }_{ij}}\right]=Tr\left[{{\varvec{\Gamma}}}^{-1}\frac{\partial{\varvec{\Gamma}}}{\partial {\gamma }_{ij}}{{\varvec{\Gamma}}}^{-1}{\mathbf{H}}_{MF}\right].$$

After some algebra that is shown in Appendix this yields:

$${\varvec{\Gamma}}={\mathbf{H}}_{MF},$$

where ${\mathbf{H}}_{MF}$ is the block of $\mathbf{H}$ corresponding to the MF with themselves.

Remember that ${\mathbf{G}}_{MF}$ is the (unobserved) genomic relationship matrix across MF, which at iteration $t$ in the E-step is “augmented” by its conditional expectation, ${\mathbf{H}}_{MF}$, the corresponding submatrix of $\mathbf{H}$. The previous expression simply says that the algorithm proceeds by updating ${\mathbf{H}}_{\left(t\right)}$ from the previous estimate ${\widehat{{\varvec{\Gamma}}}}_{\left(t-1\right)}$ and then setting ${\widehat{{\varvec{\Gamma}}}}_{\left(t\right)}\leftarrow {\mathbf{H}}_{MF(t)}$, the last being the block of ${\mathbf{H}}_{(t)}$ corresponding to MF.

#### Algorithm

1. Either $\mathbf{Z}$ where $\mathbf{Z}$ contains $\{-1,0,1\}$ readings, or $\mathbf{G}=\frac{2}{k}\mathbf{ZZ}^{\mathbf{\prime}}$ may be used. Note that $\mathbf{G}$ does not change across iterations and does not need to be full rank depending on the algorithm.
2. Use a starting value ${\widehat{{\varvec{\Gamma}}}}_{\left(t=0\right)}$ different from $\mathbf{0}$, for instance ${\widehat{{\varvec{\Gamma}}}}_{\left(t=0\right)}=0.1\mathbf{I}$.
3. Do the following steps until convergence, at iteration $t$:
  - compute $${\mathbf{H}}_{MF\left(t\right)}$$ from $${\widehat{{\varvec{\Gamma}}}}_{\left(t-1\right)}$$;
  - update: ${\widehat{{\varvec{\Gamma}}}}_{\left(t\right)}\leftarrow {\mathbf{H}}_{MF\left(t\right)}$ using one of the options below;
  - optionally, compute (exact) log-likelihood $lo{g}_{L}\left({\varvec{\Gamma}}\right)=-\left(\frac{k}{2}\right)log\left(det\left({\mathbf{A}}_{\Gamma 22}\right)\right)-\left(\frac{k}{2}\right)Tr\left({\mathbf{A}}_{\Gamma 22}^{-1}\mathbf{G}\right)$ (just for checking);
  - at convergence, ${\widehat{{\varvec{\Gamma}}}}_{\left(t\right)}$ is the estimate of $\widehat{{\varvec{\Gamma}}}$.

Convergence can be checked by comparing, for instance, the elements of the Cholesky decomposition ${\mathbf{U}}_{\left(t\right)}$, ${\mathbf{U}}_{\left(t-1\right)}$, respectively of ${{\varvec{\Gamma}}}_{\left(t\right)}$ and of ${{\varvec{\Gamma}}}_{\left(t-1\right)}$ and using $\frac{{\sum }_{i}{\sum }_{j}\left({\left({U}_{\left(t\right)}\left[i,j\right]-{U}_{\left(t-1\right)}[i,j]\right)}^{2}\right) }{{\sum }_{i}{\sum }_{j}\left({\left({U}_{\left(t-1\right)}[i,j]\right)}^{2}\right)}$. To update ${\mathbf{H}}_{MF\left(t\right)}$ there are several options:

Option 1:

1. Compute ${\mathbf{A}}_{\Gamma \left(t\right)}^{-1}$, ${\mathbf{A}}_{22\Gamma \left(t\right)}^{-1}$ from pedigree and ${\widehat{{\varvec{\Gamma}}}}_{\left(t\right)}$.
2. Compute $${\mathbf{H}}_{\left(t\right)}^{-1}={\mathbf{A}}_{\Gamma \left(t\right)}^{-1}+\left(\begin{array}{cc} \mathbf{0} & \mathbf{0} \\ \mathbf{0} & {\mathbf{G}}^{-1}-{\mathbf{A}}_{\Gamma 22\left(t\right)}^{-1}\end{array}\right)$$ for all individuals including MF. Note that $\mathbf{G}$ does not change across iterations, but ${\mathbf{A}}_{\Gamma \left(t\right)}^{-1}$ and ${\mathbf{A}}_{\Gamma 22\left(t\right)}^{-1}$ do, and so does ${\mathbf{H}}_{\left(t\right)}^{-1}$. In addition, this requires $\mathbf{G}$ to be full rank. Note that ${\mathbf{H}}_{\left(t\right)}^{-1}$ includes a square block for MF.
3. Extract the (MF, MF) block of ${\mathbf{H}}_{MF\left(t\right)}$ from ${\mathbf{H}}_{\left(t\right)}$, which can be done:
  - by repeated “solving” of ${\mathbf{H}}_{\left(t\right)}^{-1}\mathbf{x}=\mathbf{v}$, where $\mathbf{v}$ is a vector with 1 in the MF position and 0 elsewhere;
  - or by sparse inversion of ${\mathbf{H}}_{\left(t\right)}^{-1}$.

Option 2:

1. Use the expression ${{\varvec{\Gamma}}}_{t+1}={{\varvec{\Gamma}}}_{t}+{\mathbf{A}}_{\Gamma mf,2\left({\text{t}}\right)}{\mathbf{A}}_{\Gamma 22\left(t\right)}^{-1}\left(\mathbf{G}-{\mathbf{A}}_{22\Gamma \left({\text{t}}\right)}\right){\mathbf{A}}_{\Gamma 22\left(t\right)}^{-1}{\mathbf{A}}_{\Gamma 2,mf\left({\text{t}}\right)}$ [16], i.e. using the subblocks (genotyped individuals, MF) of ${\mathbf{A}}_{{\varvec{\Gamma}}}$.
2. Equivalently, use ${{\varvec{\Gamma}}}_{t+1}={{\varvec{\Gamma}}}_{t}+{\left({\mathbf{A}}_{\Gamma \left(t\right)}^{mf,mf}\right)}^{-1}{\mathbf{A}}_{\Gamma \left({\text{t}}\right)}^{mf,2} \left(\mathbf{G}-{\mathbf{A}}_{\Gamma 22\left({\text{t}}\right)}\right){\mathbf{A}}_{\Gamma \left({\text{t}}\right)}^{2,mf}{\left({\mathbf{A}}_{\Gamma \left(t\right)}^{mf,mf}\right)}^{-1}$, because ${\left({\mathbf{A}}_{\Gamma \left(t\right)}^{mf,mf}\right)}^{-1}{\mathbf{A}}_{\Gamma \left({\text{t}}\right)}^{mf,2}={\mathbf{A}}_{\Gamma mf,2\left({\text{t}}\right)}{\mathbf{A}}_{\Gamma 22\left(t\right)}^{-1}$.
3. Note that ${\mathbf{A}}_{mf,2\Gamma \left({\text{t}}\right)}={\mathbf{Q}}_{2}{{\varvec{\Gamma}}}_{t}$ for ${\mathbf{Q}}_{2}$, which is a matrix with MF proportions in genotyped individuals.

Noting that ${\mathbf{A}}_{\Gamma mf,2\left({\text{t}}\right)}{\mathbf{A}}_{\Gamma 22\left(t\right)}^{-1}\mathbf{Z}$ is a matrix which contains two times the estimates of allele frequencies, minus one [3, 17], we can put results above as a function of estimated allelic frequencies $\widehat{\mathbf{P}}$, where $\mathbf{P}$ has as many rows as markers ($k$) and as many columns as populations ($npop$), as follows:

$$\begin{aligned} {{\varvec{\Gamma}}}_{t + 1} & = {{\varvec{\Gamma}}}_{t} + {\mathbf{A}}_{{mf,2{\Gamma }\left( {\text{t}} \right)}} {\mathbf{A}}_{22\Gamma \left( t \right)}^{ - 1} \left( {{\mathbf{G}} - {\mathbf{A}}_{{22{\Gamma }\left( {\text{t}} \right)}} } \right){\mathbf{A}}_{{22{\Gamma }\left( t \right)}}^{ - 1} {\mathbf{A}}_{{2,mf{\Gamma }\left( {\text{t}} \right)}} \\ & = \left( {{{\varvec{\Gamma}}}_{t} - {\mathbf{A}}_{{mf,2{\Gamma }\left( {\text{t}} \right)}} {\mathbf{A}}_{{22{\Gamma }\left( t \right)}}^{ - 1} {\mathbf{A}}_{{2,mf{\Gamma }\left( {\text{t}} \right)}} } \right) + {\mathbf{A}}_{{mf,2{\Gamma }\left( {\text{t}} \right)}} {\mathbf{A}}_{22{\Gamma } \left( t \right)}^{ - 1} {\mathbf{GA}}_{22{\Gamma } \left( t \right)}^{ - 1} {\mathbf{A}}_{{2,mf{\Gamma }\left( {\text{t}} \right)}} \\ & = \left( {{{\varvec{\Gamma}}}_{t} - {\mathbf{A}}_{{mf,2{\Gamma }\left( {\text{t}} \right)}} {\mathbf{A}}_{{22{\Gamma }\left( t \right)}}^{ - 1} {\mathbf{A}}_{{2,mf{\Gamma }\left( {\text{t}} \right)}} } \right) \\ & \quad + \left( \frac{2}{k} \right)\left( {{\mathbf{A}}_{{mf,2{\Gamma }\left( {\text{t}} \right)}} {\mathbf{A}}_{{22{\Gamma }\left( t \right)}}^{ - 1} {\mathbf{Z}}} \right)\left( {{\mathbf{Z^{\prime}A}}_{{22{\Gamma }\left( t \right)}}^{ - 1} {\mathbf{A}}_{{2,mf{\Gamma }\left( {\text{t}} \right)}} } \right) \\ & = \left( {{{\varvec{\Gamma}}}_{t} - {\mathbf{A}}_{{mf,2{\Gamma }\left( {\text{t}} \right)}} {\mathbf{A}}_{22{\Gamma } \left( t \right)}^{ - 1} {\mathbf{A}}_{{2,mf{\Gamma }\left( {\text{t}} \right)}} } \right) \\ & \quad + \left( \frac{2}{k} \right)\left( {2{\widehat{\mathbf{P}}}_{{{\Gamma }\left( t \right)}} - \mathbf{1}_{npop} \mathbf{1}_{k}^{\prime} } \right)\left( {2{\widehat{\mathbf{P}}}_{{{\Gamma }\left( t \right)}} - \mathbf{1}_{npop} \mathbf{1}_{k}^{\prime} } \right)^{\prime} \\ \end{aligned}$$

This yields one extra option:

Option 3:

1. Use ${{\varvec{\Gamma}}}_{t+1}=\left({{\varvec{\Gamma}}}_{t}-{\mathbf{A}}_{mf,2\Gamma \left({\text{t}}\right)}{\mathbf{A}}_{22\Gamma \left(t\right)}^{-1}{\mathbf{A}}_{2,mf\Gamma \left({\text{t}}\right)}\right)+\left(\frac{2}{k}\right)\left(2{\widehat{\mathbf{P}}}_{\Gamma \left(t\right)}-{\mathbf{1}}_{npop}{\mathbf{1}}_{k}^{\prime}\right){\left(2{\widehat{\mathbf{P}}}_{\Gamma \left(t\right)}-{\mathbf{1}}_{npop}{\mathbf{1}}_{k}^{\prime}\right)}^{\prime}$ where ${\widehat{\mathbf{P}}}_{\Gamma \left(t\right)}$ contains the current estimate of all allele frequencies across populations using the current value of ${\mathbf{A}}_{{\varvec{\Gamma}}}$ (in this case $\mathbf{G}$ is not needed).
2. Equivalently, use ${{\varvec{\Gamma}}}_{t+1}={\left({\mathbf{A}}_{\Gamma \left(t\right)}^{mf,mf}\right)}^{-1}+\left(\frac{2}{k}\right)\left(2{\widehat{\mathbf{P}}}_{\Gamma \left(t\right)}-{\mathbf{1}}_{npop}{\mathbf{1}}_{k}^{\prime}\right){\left(2{\widehat{\mathbf{P}}}_{\Gamma \left(t\right)}-{\mathbf{1}}_{npop}{\mathbf{1}}_{k}^{\prime}\right)}^{\prime}$, where ${\mathbf{A}}_{\Gamma \left(t\right)}^{mf,mf}$ is the block of ${\mathbf{A}}_{\Gamma \left(t\right)}^{-1}$ e.g. that is set up using Henderson’s rules corresponding to MF, which then has to be inverted. This is because ${\left({\mathbf{A}}_{\Gamma \left(t\right)}^{mf,mf}\right)}^{-1}=\left({{\varvec{\Gamma}}}_{t}-{\mathbf{A}}_{mf,2\Gamma \left({\text{t}}\right)}{\mathbf{A}}_{22\Gamma \left(t\right)}^{-1}{\mathbf{A}}_{2,mf\Gamma \left({\text{t}}\right)}\right)$.

### Methods to estimate $\boldsymbol{\Gamma }$ across and within breeds using the increase in relationships

The previous methods do not apply well to populations, such as ruminant species with unknown parent groups defined by year of birth within breed and sometimes within sexes or selection paths. It is often the case that there are too many of these groups or they are too far away (in time, or to be more specific, in number of meiosis) from genotypes to be estimated accurately.

Here, we show how the previous method for estimating MF parameters for separate populations by pseudo-EM combines with previous work [8, 10] on how to estimate MF structured by year of birth, with the same objectives (although different methods) than Kudinov et al. [6, 9]. First, we model the change in relationship across individuals with time. Then, we plug-in the methods from the previous sections.

For a closed population, the change of mean in time can be expressed as ${\mu }_{t}={\mu }_{t-1}+{\epsilon }_{t}$, when $Var\left(\epsilon \right)$ is described by coancestry, this leads to the expression [18]:

$$\begin{aligned} Var\left( {\begin{array}{*{20}c} {\begin{array}{*{20}c} {\mu_{o} } \\ {\mu_{1} } \\ {\mu_{2} } \\ \end{array} } \\ {\mu_{3} } \\ \ldots \\ \end{array} } \right) & = \left[ {\begin{array}{*{20}c} {\overline{A}_{0} } & {\overline{A}_{0} } & {\overline{A}_{0} } & {\overline{A}_{0} } & \ldots \\ {\overline{A}_{0} } & {\overline{A}_{1} } & {\overline{A}_{1} } & {\overline{A}_{1} } & \ldots \\ {\overline{A}_{0} } & {\overline{A}_{1} } & {\overline{A}_{2} } & {\overline{A}_{2} } & \ldots \\ {\overline{A}_{0} } & {\overline{A}_{1} } & {\overline{A}_{2} } & {\overline{A}_{3} } & \ldots \\ \ldots & \ldots & \ldots & \ldots & \ldots \\ \end{array} } \right] \\ & = \left[\begin{array}{ccccc}{\overline{A} }_{0}& {\overline{A} }_{0}& {\overline{A} }_{0}& {\overline{A} }_{0}& \dots \\ {\overline{A} }_{0}& {\overline{A} }_{0}+\Delta {\overline{A} }_{1}& {\overline{A} }_{0}+\Delta {\overline{A} }_{1}& {\overline{A} }_{0}+\Delta {\overline{A} }_{1}& \dots \\ {\overline{A} }_{0}& {\overline{A} }_{0}+\Delta {\overline{A} }_{1}& {\overline{A} }_{0}+\Delta {\overline{A} }_{1}+\Delta {\overline{A} }_{2}& {\overline{A} }_{0}+\Delta {\overline{A} }_{1}+\Delta {\overline{A} }_{2}& \dots \\ {\overline{A} }_{0}& {\overline{A} }_{0}+\Delta {\overline{A} }_{1}& {\overline{A} }_{0}+\Delta {\overline{A} }_{1}+\Delta {\overline{A} }_{2}& {\overline{A} }_{0}+\Delta {\overline{A} }_{1}+\Delta {\overline{A} }_{2}+\Delta {\overline{A} }_{3}& \dots \\ \dots & \dots & \dots & \dots & \dots \end{array}\right] \end{aligned}$$

and so on. This is simply the covariance structure of the process (similar but not identical to an autoregressive process):

$${\mu }_{t}={\mu }_{t-1}+{\epsilon }_{t},$$

$$Var\left({\mu }_{0}\right)={\overline{A} }_{0,}$$

$$Var\left({\epsilon }_{t}\right)=\Delta {\overline{A} }_{t},$$

Note that, because inbreeding is half the relationship between the parents (and assuming mating at random), $\Delta {\overline{A} }_{t}={\overline{A} }_{t}-{\overline{A} }_{t-1}\approx 2{\overline{F} }_{t+1}-2{\overline{F} }_{t}=2\Delta {F}_{t+1}$.

Thus, we can describe ${\varvec{\Gamma}}$ in the same manner:

$${\varvec{\Gamma}}=\left[\begin{array}{ccccc}{\varGamma }_{0}& {\varGamma }_{0}& {\varGamma }_{0}& {\varGamma }_{0}& \dots \\ {\varGamma }_{0}& {\varGamma }_{0}+{\varDelta} {{\varGamma}}_{1}& {\varGamma }_{0}+{\varDelta} {\varGamma }_{1}& {\varGamma }_{0}+{\varDelta} {\varGamma }_{1}& \dots \\ {\varGamma }_{0}& {\varGamma }_{0}+{\varDelta} {\varGamma }_{1}& {\varGamma }_{0}+{\varDelta}{\varGamma }_{1}+{\varDelta} {\varGamma }_{2}& {\varGamma }_{0}+{\varDelta} {\varGamma }_{1}+{\varDelta} {\varGamma }_{2}& \dots \\ {\varGamma }_{0}& {\varGamma}_{0}+{\varDelta} {\varGamma }_{1}& {\varGamma }_{0}+{\varDelta} {\varGamma }_{1}+{\varDelta} {\varGamma }_{2}& {\varGamma}_{0}+{\varDelta} {\varGamma }_{1}+{\varDelta} {\varGamma }_{2}+{\varDelta} {\varGamma }_{3}& \dots \\ \dots & \dots & \dots & \dots & \dots \end{array}\right].$$

We will obtain those elements from pedigree-based inbreeding. We use the equivalence between inbreeding “with MF” $\Delta {F}_{\upgamma }$ and inbreeding with “unrelated founders” $\Delta F$, such that $\Delta {F}_{\upgamma }=\Delta F\left(1+\frac{\gamma }{2}\right)$. Then, we consider the fact that two times average inbreeding is equal to average coancestry: $\Delta {\Gamma }_{t}=2\Delta {F}_{\gamma ,t+1}$ and we assume that $\Delta {F}_{\upgamma }$ is the same across all periods, and our MF are separated by the same time distances (this can easily be modified), leading to:

$${\varvec{\Gamma}}=\left[\begin{array}{ccccc}{\varGamma }_{0}& {\varGamma }_{0}& {\varGamma }_{0}& {\varGamma }_{0}& \dots \\ {\varGamma }_{0}& {\varGamma }_{0}+2{\varDelta} {F}_{\left(\gamma \right)}& {\varGamma }_{0}+2{\varDelta} {F}_{\left(\gamma \right)}& {\varGamma }_{0}+2{\varDelta} {F}_{\left(\gamma \right)}& \dots \\ {\varGamma }_{0}& {\varGamma }_{0}+2{\varDelta} {F}_{\left(\gamma \right)}& {\varGamma }_{0}+4{\varDelta} {F}_{\left(\gamma \right)}& {\varGamma }_{0}+4{\varDelta} {F}_{\left(\gamma \right)}& \dots \\ {\varGamma }_{0}& {\varGamma }_{0}+2{\varDelta} {F}_{\left(\gamma \right)}& {\varGamma }_{0}+4{\varDelta} {F}_{\left(\gamma \right)}& {\varGamma }_{0}+6{\varDelta} {F}_{\left(\gamma \right)}& \dots \\ \dots & \dots & \dots & \dots & \dots \end{array}\right].$$

This covariance structure can be described in matrix terms as ${\varvec{\Gamma}}={\mathbf{11}}^{\prime}{\upgamma }_{0}+\mathbf{K}{\mathbf{K}}^{\mathbf{\prime}}\Delta F\left(1+\frac{{\gamma }_{0}}{2}\right)$, where ${\gamma }_{0}$ (or ${\Gamma }_{0})$ is the self-relationship of the very first metafounder and $$\mathbf{K}=\left[\begin{array}{*{20}c} 0& 0& 0& 0& \dots \\ 1& 0& 0& 0& \dots \\ 1& 1& 0& 0& \dots \\ 1& 1& 1& 0& \dots \\ \dots & \dots & \dots & \dots & \dots \end{array}\right]$$. Therefore, we have a parametric structure for ${\varvec{\Gamma}}$ in which we need only the elements $\Delta {\Gamma }_{t}$. Note that other definitions of $\mathbf{K}$ are possible, *e.g.* with different or fractional time steps.

To consider two populations, we used the following structure:

$${\varvec{\Gamma}}=\left[\begin{array}{cccccccc}{\varGamma }_{{1,1}}& {\varGamma }_{{1,1}}& {\varGamma }_{{1,1}}& \dots & {\varGamma }_{{1,2}} & {\varGamma }_{{1,2}}& {\varGamma }_{{1,2}}& \dots \vspace*{4pt}\\ {\varGamma }_{{1,1}}& {\varGamma }_{{1,1}}+2\Delta {F}^{\left(1\right)}(1-{\varGamma }_{{1,1}})& {\varGamma }_{{1,1}}+2\Delta {F}^{\left(1\right)}(1-{\varGamma }_{{1,1}})& \dots & {\varGamma }_{{1,2}} & {\varGamma }_{{1,2}}& {\varGamma }_{{1,2}}& \dots \vspace*{4pt}\\ {\varGamma }_{{1,1}}& {\varGamma }_{{1,1}}+2\Delta {F}^{\left(1\right)}(1-{\varGamma }_{{1,1}})& {\varGamma }_{{1,1}}+4\Delta {F}^{\left(1\right)}(1-{\varGamma }_{{1,1}})& \dots & {\varGamma }_{{1,2}} & {\varGamma }_{{1,2}}& {\varGamma }_{{1,2}}& \dots \vspace*{4pt}\\ \dots & \dots & \dots & \dots & \dots & \dots & \dots & \dots \vspace*{4pt}\\ {\varGamma }_{{2,1}} & {\varGamma }_{{2,1}} & {\varGamma }_{{2,1}} & \dots & {\varGamma }_{{2,2}} & {\varGamma }_{{2,2}} & {\varGamma }_{{2,2}} & \dots \vspace*{4pt}\\ {\varGamma }_{{2,1}}& {\varGamma }_{{2,1}}& {\varGamma }_{{2,1}}& \dots & {\varGamma }_{{2,2}} & {\varGamma }_{{2,2}}+2\Delta {F}^{\left(2\right)}(1-{\varGamma }_{{2,2}})& {\varGamma }_{{2,2}}+2\Delta {F}^{\left(2\right)}(1-{\varGamma }_{{2,2}})& \dots \vspace*{4pt}\\ {\varGamma }_{{2,1}}& {\varGamma }_{{2,1}}& {\varGamma }_{{2,1}}& \dots & {\varGamma }_{{2,2}} & {\varGamma }_{{2,2}}+2\Delta {F}^{\left(2\right)}(1-{\varGamma }_{{2,2}})& {\varGamma }_{{2,2}}+4\Delta {F}^{\left(2\right)}(1-{\varGamma }_{{2,2}})& \dots \vspace*{4pt}\\ \dots & \dots & \dots & \dots & \dots & \dots & \dots & \dots \end{array}\right].$$

The extension to more populations (e.g. breeds, countries, pathways of selection or combinations thereof) is immediate i.e. $${\varvec{\Gamma}}=\mathbf{X}{{\varvec{\Gamma}}}_{\mathbf{0}}{\mathbf{X}}^{{\prime}}+\mathbf{K}\left[\begin{array}{ccc}\mathbf{I} {\varDelta} {F}_{\left(\gamma \right)}^{\left(1\right)}& & \\ & \mathbf{I}\varDelta {F}_{\left(\gamma \right)}^{\left(2\right)}& \\ & & \dots \end{array}\right]\mathbf{K}^{\prime}$$, with $\mathbf{X}$ and $\mathbf{K}$ defined appropriately. If there are $n$ populations, the model needs $n$ values of $\Delta F$ and $n(n+1)/2$ values in ${{\varvec{\Gamma}}}_{\mathbf{0}}$. It is even possible to consider crossbred cases, e.g. using $\Delta {\Gamma }_{{1,2}}$ elements. Finally, we fit this structure into the pseudo-EM described before. The algorithm (which we call “pseudo-EM with $\Delta F$”) is similar to the pseudo-EM above with the following modifications:

1. Start with values of ${\varvec{\Gamma}}$ of the oldest MF only (in the two breeds example above, ${\varGamma }_{{1,1}}$, ${\varGamma }_{{1,2}}$, ${\varGamma }_{{2,2}}$). Expand them to full ${\varvec{\Gamma}}$ using the function of $\Delta F$.
2. Obtain matrix ${\mathbf{H}}_{MF\left(t\right)}$ as described in one of the three options before, as a function of ${\mathbf{A}}_{{\varvec{\Gamma}}({\text{t}})}$ and observed $\mathbf{G}$. Pick up the elements corresponding to the oldest MF, i.e., ${\varGamma }_{{1,1}}$, ${\varGamma }_{{1,2}}$, ${\varGamma }_{{2,2}}$. These are the new values. From them, expand to the whole matrix ${\varvec{\Gamma}}$ as above.

### Numerical example

This is an example of the pseudo-EM algorithm. We took the two metafounders and 12 animals in [1] with a simulated matrix $$\mathbf{G}=\left(\begin{array}{cccc}1.16 & 0.16 & 0.96 & 0.36 \\ 0.16 & 1.18 & 0.69 & 0.96 \\ 0.96 & 0.69 & 1.80 & 0.87 \\ 0.36 & 0.96 & 0.87 & 0.96\end{array}\right)$$ and starting value ${\varvec{\Gamma}}=0.1\mathbf{I}$. Pedigree is sorted so that MF precede real individuals. The first iteration yields:

$${\mathbf{H}}_{MF\left(0\right)}=\left(\begin{array}{cccccccccccccc}0.103 & 0.015 & 0.093 & 0.119 & 0.074 & 0.015 & 0.08 & 0.096 & 0.122 & 0.048 & 0.089 & 0.061 & 0.085 & 0.075 \\ 0.015 & 0.105 & 0.027 & 0.07 & 0.073 & 0.105 & 0.123 & 0.06 & 0.115 & 0.114 & 0.111 & 0.093 & 0.066 & 0.102 \\ 0.093 & 0.027 & 1.019 & 0.058 & 0.001 & 0.027 & 0.242 & 0.515 & 0.018 & 0.135 & 0.219 & 0.068 & 0.258 & 0.143 \\ 0.119 & 0.07 & 0.058 & 1.178 & 0.245 & 0.07 & 0.564 & 0.557 & 0.886 & 0.317 & 0.599 & 0.281 & 0.401 & 0.44 \\ 0.074 & 0.073 & 0.001 & 0.245 & 1.166 & 0.073 & 0.156 & 0.05 & 0.956 & 0.114 & 0.356 & 0.64 & 0.608 & 0.498 \\ 0.015 & 0.105 & 0.027 & 0.07 & 0.073 & 1.054 & 0.123 & 0.06 & 0.115 & 0.589 & 0.111 & 0.331 & 0.066 & 0.221 \\ 0.08 & 0.123 & 0.242 & 0.564 & 0.156 & 0.123 & 1.188 & 0.565 & 0.689 & 0.655 & 0.956 & 0.405 & 0.36 & 0.68 \\ 0.096 & 0.06 & 0.515 & 0.557 & 0.05 & 0.06 & 0.565 & 0.956 & 0.347 & 0.313 & 0.539 & 0.181 & 0.503 & 0.36 \\ 0.122 & 0.115 & 0.018 & 0.886 & 0.956 & 0.115 & 0.689 & 0.347 & 1.81 & 0.402 & 0.867 & 0.679 & 0.651 & 0.773 \\ 0.048 & 0.114 & 0.135 & 0.317 & 0.114 & 0.589 & 0.655 & 0.313 & 0.402 & 1.096 & 0.533 & 0.605 & 0.213 & 0.569 \\ 0.089 & 0.111 & 0.219 & 0.599 & 0.356 & 0.111 & 0.956 & 0.539 & 0.867 & 0.533 & 0.966 & 0.444 & 0.447 & 0.705 \\ 0.061 & 0.093 & 0.068 & 0.281 & 0.64 & 0.331 & 0.405 & 0.181 & 0.679 & 0.605 & 0.444 & 1.109 & 0.411 & 0.777 \\ 0.085 & 0.066 & 0.258 & 0.401 & 0.608 & 0.066 & 0.36 & 0.503 & 0.651 & 0.213 & 0.447 & 0.411 & 1.042 & 0.429 \\ 0.075 & 0.102 & 0.143 & 0.44 & 0.498 & 0.221 & 0.68 & 0.36 & 0.773 & 0.569 & 0.705 & 0.777 & 0.429 & 1.195 \end{array}\right),$$

from which the upper left 2 × 2 block, which corresponds to the two MF, is the new estimate $${\widehat{{\varvec{\Gamma}}}}_{1}=\left(\begin{array}{cc}0.103& 0.015\\ 0.015& 0.105\end{array}\right)$$. After 14 iterations, $$\widehat{{\varvec{\Gamma}}}=\left(\begin{array}{cc}0.408& 0.367\\ 0.367& 0.412\end{array}\right)$$.

## Tests

### Maximum likelihood with one metafounder

We used 29,138 genotyped animals of Lacaune dairy sheep [10]. For the sake of experimentation, we considered a single MF. Matrices $\mathbf{G}$ and ${\mathbf{A}}_{22}$ were constructed and written to disk. Then, a Julia program: (1) got ML estimates using the cubic equation described above and (2) did a one-dimensional grid search of the likelihood from functions of $\mathbf{G}$ and ${\mathbf{A}}_{22}$ as detailed in Appendix. The outcome of the program was estimates of $\gamma$ and an exploration of the log-likelihood $l(\gamma )$ curve.

**Fig. 1:**
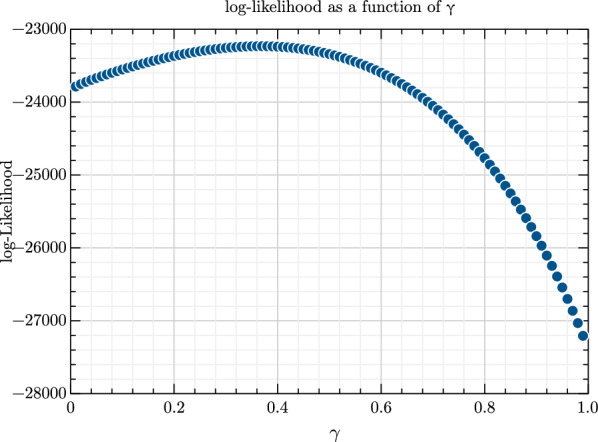
Log-likelihood of genotypes as a function of γ

Both the cubic equation and the one-dimensional grid search agreed on an ML estimate $\widehat{\gamma }=0.37$, however this value does not need to be taken seriously because the pedigree is *not* complete and therefore more than one MF should be used. The cubic equation had only one real solution. The shape of the likelihood is shown in Fig. 1. The likelihood was reasonably peaked, and using a quadratic approximation of the information matrix gives an asymptotic standard error of 0.0755.

### Simulation

We did three simulations. First, two “mixture” simulations (similar to, e.g. a synthetic breed or the introduction of US Holstein into European Friesian) with complete pedigree up to each parental breed. In the “mixture” simulations, we considered a symmetrical case (both breeds have the same genetic drift) and an asymmetrical case (breeds have different genetic drifts) (see details below).

Second, a more complex scenario of “two breeds with groups per year of birth”. This is similar to the current use of dairy cattle where most animals are purebreds but some are crossbreds, e.g. Jersey and Holstein, Nordic dairy cattle breeds, and it is similar to some sheep breeds where crossbreeding is frequent. This scenario includes two mildly related breeds.

In the “mixture, symmetrical” case, using the program macs [19], we simulated two “cattle” populations of $Ne=300$ which split 50 generations ago, resulting in 353,850 polymorphisms with a value of ${F}_{ST}=0.082$. We selected a subset of 40K loci that were polymorphic in both breeds (with a minor allele frequency (MAF) > 0.01 in both breeds) to declare them “single nucleotide polymorphisms” (SNPs) with a value of ${F}_{ST}=0.079$ (for the 40K SNPs). With these SNPs, we computed $${\varvec{\Gamma}}=\left(\begin{array}{cc}0.75& 0.64\\ 0.64& 0.75\end{array}\right)$$. The SNPs were aggregated into 30 chromosomes of 1 Morgan each. Then, we gene dropped the 40K SNPs in a complex pedigree of 10 generations with 84,200 individuals (200 sires and 4000 dams founders, followed by 10 more generations, with a progeny size of 2), completed at the top with the two MF. Individuals in the first generation were assigned to a breed origin at random with equal probability. Then, matings proceed at random, e.g. the second generation had random proportions (25/50/25) of purebreds and F1 individuals, the third generation has purebreds, F1 and F2 individuals and probably some backcrosses. For instance, looking at animals with “first breed” proportions of 0, 0.25, 0.50, 0.75, 1, each proportion had respectively an animal count of 445, 2092, 3021, 1974 and 468. Eventually, the population becomes a mixture of the two breeds; in the last generation, the proportions of breed 1 oscillate between 0.46 and 0.56. This makes estimation of ${\varvec{\Gamma}}$ more challenging as time advances. There were only two MF corresponding to the two breeds.

Then, the individuals in this complex pedigree were “genotyped” in two different ways that constitute two scenarios. The first scenario (“all generations”) consists in genotyping every 10th animal, i.e. all generations are represented with 8400 genotyped animals. The second scenario (“last generations”) considers the last 2000 of these 8400 animals, i.e. it considers animals of generations 9 to 11.

In the “mixture, unsymmetrical case”, we simulated that, after the split of populations, the $Ne$ of population 1 was 450, whereas the $Ne$ of population 2 was 45, leading to $${\varvec{\Gamma}}=\left(\begin{array}{cc}0.58& 0.25\\ 0.25& 0.65\end{array}\right)$$, with all other settings being identical. Again, there were only two MF.

**Fig. 2:**
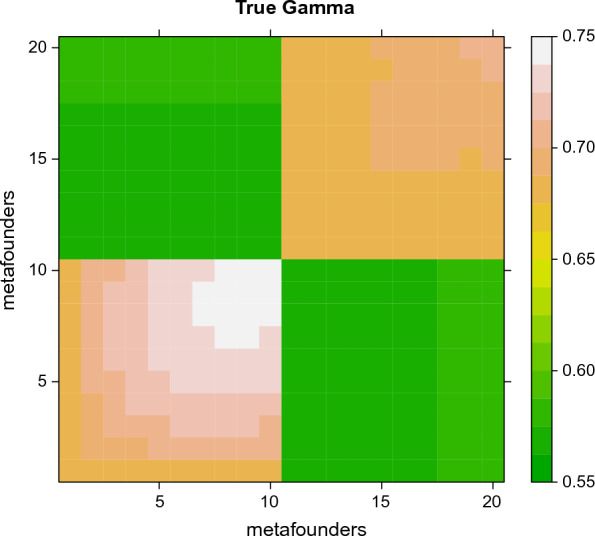
Simulated gamma for the “two breeds with groups per year of birth” scenario. The lower left block is one breed and the upper left block is another breed. Metafounders are defined every 3 years within breed

In the “two breeds with groups per year of birth” scenario, the coalescent simulation was as above, using program macs, with the initial $${\varvec{\Gamma}}=\left(\begin{array}{cc}0.68& 0.57\\ 0.57& 0.68\end{array}\right)$$. Then, we plugged the true complex pedigree of dairy sheep Latxa Cara Negra (LCN) and Manech Tête Noire (MTN) [20] with 220 K individuals spanning 30 years and ~ 10 generations (e.g. periods of 3 years), which were mainly purebreds with a few sporadic crosses (474 F1 animals, of which 58 rams with at least 10 daughters 1/4 MTN and 3/4 LCN), with missing parentships in all generations (25% missing sires and 9% missing dams after the initial generation), as it happens in real ruminant populations. There were 20 MF, 10 per breed distributed every three years (i.e. 1 to 10 for breed 1 and 11 to 20 for breed 2). We “gene dropped” markers, in 25 chromosomes of 1 Morgan each, through the pedigree. For animals in the two earliest MF (1 and 11, respectively for each breed) alleles at markers were drawn at random from corresponding allele frequencies. However, for animals in subsequent generations with missing parents, each missing parent was sampled from contemporary animals. After the simulation, we computed allele frequencies for each of the 10 generations within each of the two breeds, and we obtained true ${\varvec{\Gamma}}$ of size 20 × 20 from the cross-product ${\Gamma }_{b,{b}^{\prime}}=\frac{2}{k}\left(2{\mathbf{p}}_{b}-\mathbf{1}\right)\left(2{\mathbf{p}}_{{b}^{\prime}}-\mathbf{1}\right)^{\prime}$ with ${\mathbf{p}}_{b}$ and ${\mathbf{p}}_{{b}^{\prime}}$ being row vectors, as shown in Fig. 2. Again, the scenario “all generations” considered 10% genotyped animals across all generations, for a total of 22,433 animals, and we also considered a “last generations” scenario of 2000 animals corresponding (roughly) to the last three generations.

We applied the generalized least squares (GLS) algorithm [3] and the pseudo-EM algorithm (the stopping criterion was ${10}^{-6}$) to all these scenarios. The GLS algorithm was applied in its raw form, e.g. there was no correction for estimates of allele frequencies or of ${\varvec{\Gamma}}$ that were outside the boundaries, and also, we did not attempt a GLS+$\Delta F$ method. In the “two breeds with groups per year of birth” case, we used the model pseudo-EM+$\Delta F$, using literature values of pedigree-based $\Delta F$ of 0.0021 and 0.0016 per year for MTN and LCN [21, 22], and a time interval of 3 years between each MF. Note that although we do not use genotypes from either of these breeds, we do use their real pedigrees and hence the literature estimates are adequate.

### Results from simulation

Table 1 shows the results with the “mixture” case. When genotyped animals are present in all generations, both GLS and pseudo-EM estimate ${\varvec{\Gamma}}$ correctly. However, when information is available only for the last generations, GLS tends to overestimate the diagonal of ${\varvec{\Gamma}}$, because errors in the estimate of allele frequencies, when squared, cumulate in the diagonal. Regarding the values of the off-diagonal elements of ${\varvec{\Gamma}}$, they tend to be underestimated because errors across two MF tend to cancel out, i.e. ${\widehat{p}}_{b}{\widehat{p}}_{{b}^{\prime}}<{p}_{b}{p}_{{b}^{\prime}}$.

**Table 1:** True (simulated) and estimates of gamma using GLS or pseudo-EM and using animals from all generations (“all”) or from the last two generations (“last”)

	Symmetrical					Asymmetrical				
	True	All		Last		True	All		Last	
		GLS	Pseudo-EM	GLS	Pseudo-EM		GLS	Pseudo-EM	GLS	Pseudo-EM
${\Gamma }_{{1,1}}$	0.75	0.75	0.74	1.07	0.76	0.58	0.58	0.58	0.96	0.58
${\Gamma }_{{1,2}}$	0.64	0.63	0.64	0.31	0.64	0.25	0.24	0.25	− 0.13	0.25
${\Gamma }_{{2,2}}$	0.75	0.75	0.74	1.09	0.72	0.65	0.65	0.65	1.06	0.64

**Fig. 3:**
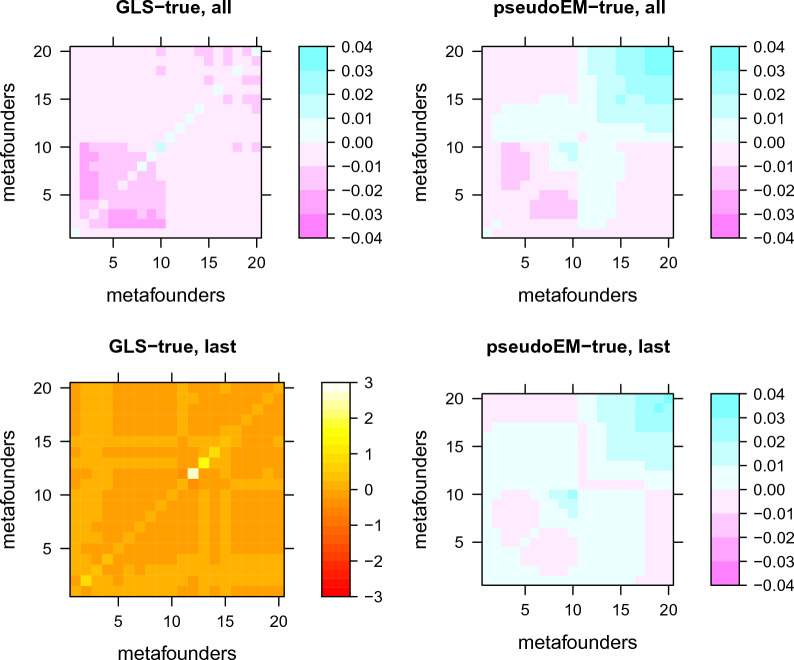
Difference between estimated and true gamma when genotyped animals are distributed in “all” generations or in the three “last” generations. Simulation “two breeds with groups per year of birth”

Results of “two breeds with groups per year of birth” scenario are in Table 2 and Figs. 2 and 3. Figure 2 shows that the true, simulated relationships in ${\varvec{\Gamma}}$ are structured within- and across-populations, and there is a slow increase in ${\varvec{\Gamma}}$ due to increased coancestry within breed. Values go from 0.69 to 0.74 (MTN) or 0.70 (LCN) within breed, and are ~ 0.57 across breeds. The simulated ${\varvec{\Gamma}}$ increases with time as expected due to increased coancestry within the breed.

**Table 2:** Statistics of true and estimated values of gamma for the “two breeds with groups per year of birth” scenario

	All		Last	
	GLS	Pseudo-EM+$\Delta F$	GLS	Pseudo-EM+$\Delta F$
Correlation, diagonal	0.967	0.783	− 0.448	0.897
Correlation, off-diagonal	0.998	0.990	0.914	0.996
Median (estimator-true), diagonal	0.000	0.007	0.045	0.009
Median (estimator-true), off-diagonal	− 0.007	0.000	− 0.005	0.002
Maxdiff (estimator-true), diagonal	0.016	0.036	2.542	0.031
Maxdiff (estimator-true), off-diagonal	− 0.022	0.033	− 0.278	0.029

Maxdiff is the maximum absolute difference, with sign

Table 2 and Fig. 3 (note that in Fig. 3 the scales differ for each panel) show the performance of the estimates. In the “all generations genotyped” scenario, both GLS and pseudo-EM+$\Delta F$ are very accurate. GLS underestimates off-diagonal relationships in the first breed, because (again) estimated allele frequencies are not perfect, whereas pseudo-EM+$\Delta F$ overestimates them in the second breed, probably because the literature estimates of $\Delta F$ that we used do not consider correctly missing pedigrees, which is shown by the bias being larger for the block that would correspond to LCN (upper right) that has more missing pedigree. In any case, both GLS and pseudo-EM+$\Delta F$ estimates of ${\varvec{\Gamma}}$ should perform adequately for genetic evaluations because the differences with the true ${\varvec{\Gamma}}$ are very small.

When only “last” generations have individuals with genotypes, GLS does not provide a good estimate because, although it does capture the block structure of the two populations, it has values that are too high in the diagonal, exceeding the biological limits of 2. On the contrary, pseudo-EM+$\Delta F$ obtains values that are quite close to true values, even if there are always small biases towards the ends. It is also worth mentioning that pseudo-EM errors are of the same (small) magnitude in the “all” and “last” scenarios, which is not true for GLS, that has a performance that is strongly affected by distance of the estimated MF to genotyped individuals.

Convergence to the required value was fast, about ~ 7 iterations for all simulations, except for the “asymmetrical, last generations genotyped” scenario, which took 59 iterations.

In all three simulations, we genotyped every 10th individual within each genotyped generation, which ensures a homogeneous genotyping. This is not true in real life where elite animals and lines are over-represented in the genotypes, which might lead to biases in the estimation of ${\varvec{\Gamma}}$.

## Discussion

The lack of a general method to estimate ${\varvec{\Gamma}}$ is a real problem for use of MF in genetic evaluations. Indeed, several studies used ad hoc techniques to estimate ${\varvec{\Gamma}}$, e.g. [6, 8] and the lack of a general method is a frequent complaint. In our experience, the simple GLS method yields some parts of ${\varvec{\Gamma}}$ that are well estimated (i.e. for large breeds) whereas other parts are not (e.g. for small breeds).

Our method of ML for one population is rather simple and not more expensive computationally than the GLS method. Although a bit more complex to implement, the ML method provides estimates that are guaranteed to be in the parametric space and more robust than the GLS method estimates. For the cases where there are no genetic groups or MF, Garcia-Baccino et al. [3] proved that using a single MF was better than the current methods of “tuning” the $\mathbf{G}$ matrix [23], and that the ML method or the GLS method provided good estimates. Another option is, of course, to use base allele frequencies to build $\mathbf{G}$.

Our methods of pseudo-EM and pseudo-EM+$\Delta F$ do a good job, in particular when information is “asymmetrical” or genotypes exist only in the last generations. Our method uses already available $\Delta F$ based on pedigree. It may be argued that $\Delta F$ changes with time, but past $\Delta F$ does not change, and the method can easily accommodate different $\Delta F$ along time by changing $\mathbf{K}$, $\Delta F$, or both. Estimation of $\Delta F$ is challenging in itself [7, 24] and underestimation of $\Delta F$ will result in values of ${\varvec{\Gamma}}$ too close to each other. In our sheep-based example, we have not applied any particular technique for correction of missing pedigree and the results are reasonable, which seems to imply that the method is robust to a small number of missing pedigree records.

An alternative method by Kudinov et al. [6, 9] models ${\varvec{\Gamma}}$ using covariance functions in a rather general manner, i.e. it would be of the form ${\varvec{\Gamma}}={\varvec{\Phi}}{{\varvec{\Gamma}}}_{0}{{\varvec{\Phi}}}^{\prime}$ where ${\varvec{\Phi}}$ is a matrix that is similar to our $\mathbf{K}$ matrices and ${{\varvec{\Gamma}}}_{0}$ can be estimated from data. This method is expected to properly describe the increase in coancestry in closed populations, provided enough information (genotypes) across time is given, which may not be true in all cases, for instance, for beef or sheep. It is possibly of interest to dig into the similarities of the two methods and combine them.

Another alternative method in the literature is GLS to obtain allele frequencies [3, 17, 25], followed by the equation ${\widehat{\Gamma }}_{{\text{b}},{{\text{b}}}^{\prime}}=8Cov\left({\widehat{p}}_{b},{\widehat{p}}_{{b}^{\prime}}\right)$ [3]. We have improved this method in two manners. First, we use a better definition of ${{\varvec{\Gamma}}}_{b,{b}^{\prime}}=\left(\frac{2}{k}\right)\left(2{\mathbf{p}}_{b}-\mathbf{1}\right)\left(2{\mathbf{p}}_{{b}^{\prime}}-\mathbf{1}\right)^{\prime}$, where ${\mathbf{p}}_{b}$ and ${{\mathbf{p}}}_{{b}^{\prime}}$ are row vectors of allele frequencies, which does not rely on random coding of alleles. Second, the equation ${\widehat{{\varvec{\Gamma}}}}_{{\text{b}},{{\text{b}}}^{{\prime}}}=8Cov\left({\widehat{p}}_{b},{\widehat{p}}_{{b}^{\prime}}\right)$ uses estimated $\widehat{p}$ in place of true $p$ and this leads to cumulation of errors within MF (upward bias in the diagonal) or negative covariance of errors across MF (downward bias off-diagonal) globally leading to biased estimates of ${\varvec{\Gamma}}$ for extreme cases (i.e. our simulations with “last” individual genotyped). On the one hand, the GLS method can be refined and made into a sort of GLS+$\Delta F$ method [10] although we have not attempted to do this. On the other hand, the pseudo-EM strategy is not more expensive computationally and has better theoretical properties.

Compared to GLS, pseudo-EM should be a more robust method because it is an (approximation of) EM algorithm, i.e., it should yield estimates within the parametric space, as far as the approximation is a good one. Pseudo-EM+${\Delta F}$ yielded estimates within the parameter space in the case “two breeds with groups per year of birth”, whereas GLS did not. Computing times of GLS and pseudo-EM (with or without $\Delta F$) are similar if efficiently programmed because both require some form of either ${\mathbf{A}}_{\Gamma }^{-1}$ or ${\mathbf{A}}^{-1}$ and manipulation of $\mathbf{G}$ or $\mathbf{Z}$ depending on the actual form of the algorithms. A difference is that pseudo-EM is an iterative algorithm that requires some iterations, which in our case were mostly a small number ($\le 7$) but not always (59 for one case).

It is also of interest to present pseudo-EM compared to previous algorithms and true ML. Consider the update ${{\varvec{\Gamma}}}_{t+1}={\left({\mathbf{A}}_{\Gamma \left(t\right)}^{mf,mf}\right)}^{-1}+\left(\frac{2}{k}\right)\left(2{\widehat{\mathbf{P}}}_{\Gamma \left(t\right)}-{\mathbf{1}}_{npop}{\mathbf{1}}_{k}^{\prime}\right){\left(2{\widehat{\mathbf{P}}}_{\Gamma \left(t\right)}-{\mathbf{1}}_{npop}{\mathbf{1}}_{k}^{\prime}\right)}^{\prime}$. The second part of the update, $\left(\frac{2}{k}\right)\left(2{\widehat{\mathbf{P}}}_{\Gamma \left(t\right)}-{\mathbf{1}}_{npop}{\mathbf{1}}_{k}^{\prime}\right){\left(2{\widehat{\mathbf{P}}}_{\Gamma \left(t\right)}-{\mathbf{1}}_{npop}{\mathbf{1}}_{k}^{\prime}\right)}^{\prime}$, corresponds to the GLS estimator of [3], the differences being that the latter: (1) used $\mathbf{A}$ (not ${\mathbf{A}}_{\Gamma }$), (2) was not iterated and (3) used the covariance across allele frequencies instead of the cross-product. In addition, the first part of the update ${\left({\mathbf{A}}_{\Gamma \left(t\right)}^{mf,mf}\right)}^{-1}$ considers the prediction error variance in the prediction of allele frequencies in $\mathbf{P}$, i.e. the more the genotyped animals are far from MF, the less the EM algorithm relies on estimates of allele frequencies. The prediction error variance will be different for MF that have less information in genotyped animals.

This expression also allows us to see that, the left part of the update $\left({{\varvec{\Gamma}}}_{t}-{\mathbf{A}}_{mf,2\Gamma \left({\text{t}}\right)}{\mathbf{A}}_{22\Gamma \left(t\right)}^{-1}{\mathbf{A}}_{2,mf\Gamma \left({\text{t}}\right)}\right)$ is the prediction error covariance matrix of genotypes for MF given the observed $\mathbf{G}$ [15], and this prediction error covariance which was included in the approximate ML estimator suggested (but not actually used) by Garcia-Baccino et al. [3].

Matrix ${\mathbf{A}}_{2,mf\Gamma \left({\text{t}}\right)}$ in ${\widehat{{\varvec{\Gamma}}}}_{t+1}={\widehat{{\varvec{\Gamma}}}}_{t}+{\mathbf{A}}_{mf,2\Gamma \left({\text{t}}\right)}{\mathbf{A}}_{22\Gamma \left(t\right)}^{-1}\left(\mathbf{G}-{\mathbf{A}}_{22\Gamma \left({\text{t}}\right)}\right){\mathbf{A}}_{22\Gamma \left(t\right)}^{-1}{\mathbf{A}}_{2,mf\Gamma \left({\text{t}}\right)}$ is actually a matrix of breed proportions ${\mathbf{A}}_{2,mf\Gamma \left({\text{t}}\right)}={\mathbf{Q}}_{2}{\widehat{{\varvec{\Gamma}}}}_{t}$, which gives ${\widehat{{\varvec{\Gamma}}}}_{t+1}={\widehat{{\varvec{\Gamma}}}}_{t}+{\widehat{{\varvec{\Gamma}}}}_{t}{\mathbf{Q}}_{2}^{\prime}{\mathbf{A}}_{22\Gamma \left(t\right)}^{-1}\left(\mathbf{G}-{\mathbf{A}}_{22\Gamma \left({\text{t}}\right)}\right){\mathbf{A}}_{22\Gamma \left(t\right)}^{-1}{\mathbf{Q}}_{2}{\widehat{{\varvec{\Gamma}}}}_{t}$. At convergence $\widehat{{\varvec{\Gamma}}}={\widehat{{\varvec{\Gamma}}}}_{t+1}={\widehat{{\varvec{\Gamma}}}}_{t}$ which implies that $\widehat{{\varvec{\Gamma}}}$ is the solution to the (non-linear) equation $\mathbf{0}={\mathbf{Q}}_{2}^{\prime}{\mathbf{A}}_{22\Gamma \left(t\right)}^{-1}\left(\mathbf{G}-{\mathbf{A}}_{22\Gamma \left({\text{t}}\right)}\right){\mathbf{A}}_{22\Gamma \left(t\right)}^{-1}{\mathbf{Q}}_{2}$.

As for the comparison with true ML, consider this last non-linear equation $\mathbf{0}={\mathbf{Q}}_{2}^{\prime}{\mathbf{A}}_{22\Gamma \left(t\right)}^{-1}\left(\mathbf{G}-{\mathbf{A}}_{22\Gamma \left({\text{t}}\right)}\right){\mathbf{A}}_{22\Gamma \left(t\right)}^{-1}{\mathbf{Q}}_{2}$ in the case of single MF: $\mathbf{0}={\mathbf{1}}^{\prime}{\mathbf{A}}_{22\Gamma \left(t\right)}^{-1}\left({\mathbf{G}}-{\mathbf{A}}_{22\Gamma \left({\text{t}}\right)}\right){\mathbf{A}}_{22\Gamma \left(t\right)}^{-1}\mathbf{1}$. After applying the identities to ${\mathbf{A}}_{\gamma 22}={\mathbf{A}}_{22}\left(1-\frac{\gamma }{2}\right)$ and some algebra, the preceding non-linear equation yields the linear equation on $\gamma$:

$$1-\frac{\gamma }{2}+\gamma {\mathbf{1}}^{\prime}{\mathbf{A}}_{22}^{-1}\mathbf{1}=\frac{{\mathbf{1}}^{\prime}{\mathbf{A}}_{22}^{-1}\mathbf{G}{\mathbf{A}}_{22}^{-1}\mathbf{1}}{{\mathbf{1}}^{\prime}{\mathbf{A}}_{22}^{-1}\mathbf{1}}.$$

The solution of this equation is an estimate $\widehat{\gamma }$. However, we note that compared to ML, the term $Tr\left({\mathbf{A}}_{22}^{-1}\mathbf{G}\right)$ does not appear here. Therefore, the solution to this equation is not the ML estimate. We also note that for $n$ unrelated individuals ${\mathbf{A}}_{22}={\mathbf{I}}_{n}$, and we obtain $\gamma \approx \frac{1}{{n}^{2}}{\mathbf{1}}^{\prime}\mathbf{G}1=mean(\mathbf{G})$ as expected.

Last, it has to be recalled that the Mendelian sampling variances ${D}_{i,i}$ contain likelihood information about ${\varvec{\Gamma}}$, which is used in true ML but the maximization of which is unclear in pseudo-EM. For instance, F1 and F2 individuals A×B or (A×B)×(A×B), in spite of having the same breed proportions will have different Mendelian sampling variances of their respective gametes [13].

## Conclusions

The theory of MF allows a general method that accommodates pedigree and genomic relationships correctly. However, its use demands estimation of relationships across base populations (${\varvec{\Gamma}}$) which is complex, in particular in the complex pedigrees used in livestock genetics. Using Gaussian likelihoods, we derived ML, pseudo-EM and pseudo-EM+$\Delta F$ methods to estimate ${\varvec{\Gamma}}$ in many realistic settings. These methods require either set up and comparison of genomic and pedigree relationships, or use of allele frequency estimates based on observed markers and pedigree, sometimes completed with additional information (evolution of inbreeding) from pedigrees. Computational cost is therefore low. Estimates are accurate in real and simulated data. These methods will help testing and using MF for genetic evaluations in livestock species.

## Data Availability

Not applicable.

## Appendix

### Computational tricks to obtain the likelihood and parts of the maximization algorithm

#### Likelihood

The likelihood is composed of two parts, $log\left(det\left({\mathbf{A}}_{\Gamma 22}\right)\right)$ and $Tr\left({\left({\mathbf{A}}_{\Gamma 22}\right)}^{-1}\mathbf{G}\right)$. The value of $log\left(det\left({\mathbf{A}}_{\Gamma 22}\right)\right)$ can be computed as follows:

*(option 1)* get log-determinant of ${\mathbf{A}}_{\Gamma 22}$ using:

1. Compute $${\mathbf{A}}_{\Gamma 22}$$
2. $\mathbf{L}=cholesky\left({\mathbf{A}}_{\Gamma 22}\right)$ i.e., the lower triangular factor of its Cholesky decomposition.
3. $ln\left|{\mathbf{A}}_{\Gamma 22}\right|=2\sum log{L}_{ii}$ where ${L}_{ii}$ are the diagonals of the **L** Cholesky factor.

*(option 2)* $ln\left|{\mathbf{A}}_{\Gamma 22}\right|$ can be obtained from sparse matrices ${\mathbf{A}}_{\Gamma }^{-1}$ and ${\mathbf{A}}_{\Gamma }^{11}$ (the (sparse) block of the (sparse) ${\mathbf{A}}_{\Gamma }^{-1}$ corresponding to non-genotyped individuals and metafounders) using partitioned matrix theory as:

$${\text{log}}\left|{\mathbf{A}}_{\Gamma 22}\right|=log\left|{\mathbf{A}}_{\Gamma }^{11}\right|-log\left|{\mathbf{A}}_{\Gamma }^{-1}\right|,$$

where $log\left|{\mathbf{A}}_{\Gamma }^{11}\right|$ is computed e.g. using sparse inversion. The $log\left|{\mathbf{A}}_{\Gamma }^{-1}\right|$ is computed as follows. ${\mathbf{A}}_{\Gamma }^{-1}={\mathbf{T}}^{-1}{\mathbf{D}}^{-1}\mathbf{T}^{{\prime}-1}$ and therefore $\left|{\mathbf{A}}_{\Gamma }^{-1}\right|=\left|{\mathbf{T}}^{-1}\right|\left|{\mathbf{D}}^{-1}\right|\left|\mathbf{T}^{{\prime} -1}\right|=\left|{\mathbf{D}}^{-1}\right|$ because ${\mathbf{T}}^{-1}$ is a triangular matrix with a diagonal of 1 s.

Matrix $\left|{\mathbf{D}}^{-1}\right|$ contains two parts, the part linked to MF and the part linked to “animals”:

$$\left|{\mathbf{D}}^{-1}\right|=\left|{{\varvec{\Gamma}}}^{-1}\right|\times {\Pi }_{i}\frac{1}{{d}_{ii}},$$

where $1/{d}_{ii}$ are the contributions summed in Henderson’s algorithm for the **A**-inverse, but here depends on ${\varvec{\Gamma}}$. Thus:

$$log\left|{\mathbf{A}}_{\Gamma }^{-1}\right|=log\left|{{\varvec{\Gamma}}}^{-1}\right|+\sum log\left(\frac{1}{{d}_{ii}}\right),$$

where the summation extends to all animals in the pedigree.

The value of $Tr\left({\mathbf{A}}_{\Gamma 22}^{-1}\mathbf{G}\right)$ can be obtained in at least two different ways.

$$b=Tr\left({\mathbf{A}}_{\Gamma 22}^{-1}\mathbf{G}\right)={\sum }_{{\text{i}}}{\sum }_{j}\left({\left({\mathbf{A}}_{\Gamma 22}^{-1}\right)}_{ij}{\mathbf{G}}_{ij}\right),$$

in other words, as the sum of elements of the direct product ${\mathbf{A}}_{\Gamma 22}^{-1}\odot \mathbf{G}$, which is very easy if they have already been computed.

Alternatively, for instance if ${\mathbf{A}}_{\Gamma 22}^{-1}$ is not directly computed but products ${\mathbf{A}}_{\Gamma 22}^{-1}\mathbf{z}$ can be computed [26]:

$$b=Tr({\mathbf{A}}_{\Gamma 22}^{-1}\mathbf{G})=Tr\left({\mathbf{A}}_{\Gamma 22}^{-1}\mathbf{Z}{\mathbf{Z}}^{{\prime}}\frac{2}{k}\right)=\frac{2}{k} \sum {\mathbf{z}}_{j}^{{\prime}}{\mathbf{A}}_{\Gamma 22}^{-1}{\mathbf{z}}_{j},$$

where the summation includes $j=1,\dots ,k$ loci.

#### Parts of the single-metafounder exact ML equation

The value of $a=\mathbf{1}^{\prime}{\mathbf{A}}_{22}^{-1}\mathbf{1}$ can be computed as the sum of the elements of ${\mathbf{A}}_{22}^{-1}$ (if explicitly computed) or using an indirect method to yield products ${\mathbf{A}}_{\Gamma 22}^{-1}1$.

For the value of $b$, see above.

The value of $c=\mathbf{1}^{\prime}{\mathbf{A}}_{\Gamma 22}^{-1}\mathbf{G}{\mathbf{A}}_{\Gamma 22}^{-1}\mathbf{1}$ can be obtained explicitly if matrices are available or indirectly, noting that $c=\mathbf{1}^{\prime}{\mathbf{A}}_{\Gamma 22}^{-1}\mathbf{G}{\mathbf{A}}_{\Gamma 22}^{-1}\mathbf{1}={\mathbf{1}}^{{\prime}}{\mathbf{A}}_{\Gamma 22}^{-1}\left(\frac{2}{k}\mathbf{Z}{\mathbf{Z}}^{{\prime}}\right){\mathbf{A}}_{\Gamma 22}^{-1}\mathbf{1}=\frac{2}{k}\mathbf{t}^{\prime}\mathbf{t}=$ where $\mathbf{t}$ is a vector of size $k$ number of markers, $\mathbf{t}^{\prime}=\mathbf{1}^{\prime}{\mathbf{A}}_{\Gamma 22}^{-1}\mathbf{Z}$. Elements of $\mathbf{t}$ can be computed by pre-computing the vector $\mathbf{q}=\mathbf{1}^{\prime}{\mathbf{A}}_{\Gamma 22}^{-1}$ and then looping through markers as ${t}_{j}={\mathbf{q}}^{\prime}{\mathbf{z}}_{j}$ or directly, in a loop similar as above, as:

$$c=\frac{2}{k}\sum_{j}{\left({\mathbf{q}}^{\mathbf{\prime}}{\mathbf{z}}_{j}\right)}^{2}.$$

### Exact ML for one metafounder

Consider $lo{g}_{L}\left({\varvec{\Gamma}}\right)=-\left(k/2\right)log\left(det\left({\mathbf{A}}_{\Gamma 22}\right)\right)-\left(k/2\right)Tr\left({\left({\mathbf{A}}_{\Gamma 22}\right)}^{-1}\mathbf{G}\right)$. Using formulas from [27], we obtain:

$${\left({\mathbf{A}}_{\gamma 22}\right)}^{-1}={\mathbf{A}}_{22}^{-1}\left(1-\gamma /2\right)-\frac{{\mathbf{A}}_{22}^{-1}{\mathbf{11}}^{{\prime}}\gamma }{\left(\left(1-\frac{\gamma }{2}\right)\left(1-\frac{\gamma }{2}+\gamma {\mathbf{1}}^{{\prime}}{\mathbf{A}}_{22}^{-1}\mathbf{1}\right)\right)},$$

$$\begin{aligned}det\left({\mathbf{A}}_{\gamma 22}\right)& = det\left({\mathbf{A}}_{22}^{-1}\right){\left(1-\gamma /2\right)}^{n}\left(1+\mathbf{1}^{\prime}{\mathbf{A}}_{22}^{-1}\mathbf{1}/\left(1-\gamma /2\right)\right)\\ & = det\left({\mathbf{A}}_{22}^{-1}\right){\left(1-\gamma /2\right)}^{n-1}\left(1+\mathbf{1}^{\prime}{\mathbf{A}}_{22}^{-1}\mathbf{1}\right)\end{aligned}.$$

Inserting those into the log-likelihood function, and using short-hand notation $a=\mathbf{1}^{\prime}{\mathbf{A}}_{22}^{-1}\mathbf{1}$, $b=Tr\left({\mathbf{A}}_{22}^{-1}\mathbf{G}\right)$ and $c=Tr\left({\mathbf{A}}_{22}^{-1}\mathbf{11}^{\prime}{\mathbf{A}}_{22}^{-1}\mathbf{G}\right)=\mathbf{1}^{\prime}{\mathbf{A}}_{22}^{-1}\mathbf{G}{\mathbf{A}}_{22}^{-1}\mathbf{1}$, i.e. the sum of all elements of ${\mathbf{A}}_{22}^{-1}\mathbf{G}{\mathbf{A}}_{22}^{-1}$, we obtain:

$$\begin{aligned} lo{g}_{L}\left(\gamma \right)&= -\left(\frac{k}{2}\right)log\left(det\left({\mathbf{A}}_{22}\right)\right)-\left(\frac{k}{2}\right)\left(n-1\right)log\left(1-\frac{\gamma }{2}\right)-\left(\frac{k}{2}\right)log\left(1-\frac{\gamma }{2}+\gamma a\right)\\ & \quad -\left(\frac{k}{2}\right)\frac{b}{\left(1-\frac{\gamma }{2}\right)}+\left(\frac{k}{2}\right)c\frac{\gamma }{\left(1-\frac{\gamma }{2}\right)\left(1-\frac{\gamma }{2}+\gamma a\right)}. \end{aligned}$$

The ML estimate is obtained by differentiating $l\left(\gamma \right)$ and setting equal to zero, where we note that the $k/2$ in front of each term in the expression can be ignored,

$$\begin{aligned} 0 & = \frac{{\left( {n - 1} \right)/2}}{{\left( {1 - \gamma /2} \right)}} - \frac{ - 1/2 + a}{{1 - \gamma /2 + \gamma a}} - \frac{b/2}{{\left( {1 - \gamma /2} \right)^{2} }} \\ & \quad + c\frac{{\left( {1 - \frac{\gamma }{2}} \right)\left( {1 - \frac{\gamma }{2} + \gamma a} \right) - \gamma \left( {\left( {1 - \frac{\gamma }{2}} \right)\left( { - \frac{1}{2} + a} \right) - \frac{{\left( {1 - \frac{\gamma }{2} + \gamma a} \right)}}{2}} \right)}}{{\left( {1 - \gamma /2} \right)^{2} \left( {1 - \gamma /2 + \gamma a} \right)^{2} }}. \\ \end{aligned}$$

Multiplying by $\left({\left(1-\gamma /2\right)}^{2}\right){\left(1-\gamma /2+\gamma a\right)}^{2}$ on both sides of the equation followed by some algebraic manipulations, we obtain a cubic equation:

$${e}_{3}{\gamma }^{3}+{e}_{2}{\gamma }^{2}+{e}_{1}\gamma +{e}_{o}=0,$$

where $${e}_{3}=-n{\left(-1/2+a\right)}^{2}/2$$, $${e}_{2}=n\left(\left(-3/2+a\right)+a-b\left(-1/2+a\right)+c\right)\left(-1/2+a\right)$$, $${e}_{1}=\left(n-1\right)\left(-3/2+2a\right)-\left(-1+2a\right)\left(-3/2+a+b\right)$$ and $${e}_{0}=n-2a-b+2c$$.

Solving a cubic equation, first requires the computation of the discriminant $\varDelta =18{e}_{3}{e}_{2}{e}_{1}{e}_{0}-4{e}_{2}^{3}{e}_{0}+{e}_{2}^{2}{e}_{1}^{2}-4{e}_{3}{e}_{1}^{3}+27{e}_{3}^{2}{e}_{0}^{2}$. If $\varDelta >0$, then there are three distinct real roots, and if $\varDelta <0$, then there is only one real root, which is the ML estimate of $\gamma$ (if in the parametric space), and two complex roots.

### Derivatives of the likelihood

#### The first derivative of $Tr\left({\mathbf{A}}_{\Gamma }^{-1}\mathbf{G}\right)$

Based on the definition of trace, and using rules for differentiation of a trace [28]:

$$\begin{aligned} Tr\left( {{\mathbf{A}}_{{\Gamma }}^{ - 1} {\mathbf{G}}} \right) & = Tr\left( {{\mathbf{T}}^{{ - 1{\prime}}} {\mathbf{D}}_{{\Gamma }}^{ - 1} {\mathbf{T}}^{ - 1} {\mathbf{G}}} \right) = Tr\left( {{\mathbf{D}}_{{\Gamma }}^{ - 1} {\mathbf{T}}^{ - 1} {\mathbf{GT}}^{{ - 1{\prime}}} } \right) \\ & = Tr\left( {{\mathbf{D}}_{{\Gamma }}^{ - 1} {\mathbf{T}}^{ - 1} \frac{{{\mathbf{ZZ^{\prime}}}}}{\frac{k}{2}}{\mathbf{T}}^{{ - 1{\prime}}} } \right) = Tr\left( {{\mathbf{D}}_{{\Gamma }}^{ - 1} {\mathbf{WW}}^{\prime}} \right) = Tr\left( {{\mathbf{D}}_{{\Gamma }}^{ - 1} {\mathbf{B}}} \right), \\ \end{aligned}$$

where $\mathbf{W}={\mathbf{T}}^{-1}\mathbf{Z}/\sqrt{0.5k},\mathbf{B}=\mathbf{W}\mathbf{W}^{\prime}$. Note that the block of ${\mathbf{T}}^{-1}$ corresponding to MF is simply an identity matrix, and thus the block of $\mathbf{B}$ corresponding to MF is simply ${\mathbf{G}}_{MF}$. Thus,

$$\frac{\partial Tr\left({\mathbf{A}}_{\Gamma }^{-1}\mathbf{G}\right)}{\partial \gamma }=\frac{\partial Tr\left({\mathbf{D}}_{\Gamma }^{-1}\mathbf{B}\right)}{\partial \gamma }=Tr\left(\frac{\partial {\mathbf{D}}_{\Gamma }^{-1}}{\partial \gamma }\mathbf{B}\right).$$

#### The first derivative of $log\left(det\left({\mathbf{A}}_{\Gamma }\right)\right)$

Based on the definition of Jacobi formula (see for instance chap. 15, 8.5 in [28]):

$$\begin{aligned} \frac{{\partial det\left( {{\mathbf{A}}_{{\Gamma }} } \right)}}{\partial \gamma } & = det\left( {{\mathbf{A}}_{{\Gamma }} } \right)Tr\left( {{\mathbf{A}}_{{\Gamma }}^{ - 1} \frac{{\partial {\mathbf{A}}_{{\Gamma }} }}{\partial \gamma }} \right) \\ & = det\left( {{\mathbf{A}}_{{\Gamma }} } \right)Tr\left( {{\mathbf{T}}^{ - 1{\prime}}{\mathbf{D}}_{{\Gamma }}^{ - 1} {\mathbf{T}}^{ - 1} \frac{{\partial {\mathbf{A}}_{{\Gamma }} }}{\partial \gamma }} \right) \\ & = det\left( {{\mathbf{A}}_{{\Gamma }} } \right)Tr\left( {{\mathbf{T}}^{ - 1{\prime}}{\mathbf{D}}_{{\Gamma }}^{ - 1} {\mathbf{T}}^{ - 1} {\mathbf{T}}\frac{{\partial {\mathbf{D}}_{{\Gamma }} }}{\partial \gamma }{\mathbf{T}}^{\prime}} \right), \\ & = det\left( {{\mathbf{A}}_{{\Gamma }} } \right)Tr\left( {{\mathbf{T}}^{\prime}{\mathbf{T}}^{ - 1{\prime}}{\mathbf{D}}_{{\Gamma }}^{ - 1} {\mathbf{T}}^{ - 1} {\mathbf{T}}\frac{{\partial {\mathbf{D}}_{{\Gamma }} }}{\partial \gamma }} \right) \\ & = det\left( {{\mathbf{A}}_{{\Gamma }} } \right)Tr\left( {{\mathbf{D}}_{{\Gamma }}^{ - 1} \frac{{\partial {\mathbf{D}}_{{\Gamma }} }}{\partial \gamma }} \right) \\ \end{aligned}$$

where we note that $\partial {D}_{\varGamma }/\partial \gamma$ depends on which parameter the function is differentiated with respect to, but it does not depend on the actual values of any of the parameters. Therefore,

$$\frac{\partial log\left(det\left({\mathbf{A}}_{\Gamma }\right)\right)}{\partial \gamma }=\frac{1}{det\left({\mathbf{A}}_{\Gamma }\right)}\frac{\partial det\left({\mathbf{A}}_{\Gamma }\right)}{\partial \gamma }=Tr\left({\mathbf{D}}_{\Gamma }^{-1}\frac{\partial {\mathbf{D}}_{\Gamma }}{\partial \gamma }\right).$$

#### The first derivative of *logL*(${\varvec{\Gamma}}$)

Based on the preceding equations, the first derivative of log-likelihood function would be:

$$\begin{aligned} \frac{{\partial log_{L} \left( {{\varvec{\Gamma}}} \right)}}{\partial \gamma } & = - \frac{k}{2}\left[ {\frac{{\partial log\left( {det\left( {{\mathbf{A}}_{{\Gamma }} } \right)} \right)}}{\partial \gamma } + \frac{{\partial Tr\left( {{\mathbf{A}}_{{\Gamma }}^{ - 1} {\mathbf{G}}} \right)}}{\partial \gamma }} \right] \\ & = - \frac{k}{2}\left[ {Tr\left( {{\mathbf{D}}_{{\Gamma }}^{ - 1} \frac{{\partial {\mathbf{D}}_{{\Gamma }} }}{\partial \gamma }} \right) + Tr\left( {\frac{{\partial {\mathbf{D}}_{{\Gamma }}^{ - 1} }}{\partial \gamma }{\mathbf{B}}} \right)} \right]. \\ & = - \frac{k}{2}\left[ {Tr\left( {\frac{{\partial {\mathbf{D}}_{{\Gamma }}^{ - 1} }}{\partial \gamma }\left( {{\mathbf{B}} - {\mathbf{D}}_{{\Gamma }} } \right)} \right)} \right] \\ \end{aligned}$$

The structure of $\mathbf{B}$ and ${\mathbf{D}}_{{\varvec{\Gamma}}}$ is:

$${\mathbf{D}}_{{\varvec{\Gamma}}}=\left\{\begin{array}{ll}{\varvec{\Gamma}},& \text{for}\; \text{metafounders}\\ \text{diagonal}\; \text{matrix},& \text{for}\; \text{non-metafounders}\end{array},\right.$$

$$\mathbf{B}=\left\{\begin{array}{ll}{\mathbf{G}}_{MF},& \text{for}\; \text{metafounders}\\ {\mathbf{W}}_{NMF}{\mathbf{W}}_{NMF}^{\prime},& \text{for}\; \text{non-metafounders}\end{array}\right..$$

Thus, $\frac{\partial lo{g}_{L}\left({\varvec{\Gamma}}\right)}{\partial \gamma }$ can be expressed as follows:

$$\frac{\partial {{\text{log}}}_{L}\left({\varvec{\Gamma}}\right)}{\partial \gamma }=-\frac{k}{2}\left[Tr\left[\frac{\partial {{\varvec{\Gamma}}}^{-1}}{\partial \gamma }\left({\mathbf{G}}_{MF}-{\varvec{\Gamma}}\right)\right]\right]-\frac{k}{2}\left[\sum_{i}\left[\frac{\partial {D}_{{\varGamma }_{i,i}}^{-1}}{\partial \gamma }\left({B}_{i,i}-{D}_{{\varGamma }_{i,i}}\right)\right]\right],$$

where sum over $i$ is for non-metafounders and $\frac{\partial {{\varvec{\Gamma}}}^{-1}}{\partial \gamma }=-{{\varvec{\Gamma}}}^{-1}\frac{\partial{\varvec{\Gamma}}}{\partial \gamma }{{\varvec{\Gamma}}}^{-1}$ and $\frac{\partial {{D}_{\varGamma }^{-1}}_{ii}}{\partial \gamma }=-\frac{\partial {{D}_{\varGamma }}_{ii}}{\partial \gamma }/{\left({{D}_{\varGamma }}_{i,i}\right)}^{2}$. Inserting this, we obtain:

$$\frac{\partial {{\text{log}}}_{L}\left({\varvec{\Gamma}}\right)}{\partial \gamma }=-\frac{k}{2} \left[Tr\left[{{\varvec{\Gamma}}}^{-1} \frac{\partial{\varvec{\Gamma}}}{\partial \gamma } \left(\mathbf{I}-{{\varvec{\Gamma}}}^{-1} {\mathbf{G}}_{MF} \right)\right]\right]-\frac{k}{2}\left[\sum_{i}\left[\frac{\partial {D}_{{\varGamma }_{i,i}}}{\partial \gamma }\left(\frac{1}{{D}_{{\varGamma }_{i,i}}}-\frac{{B}_{i,i}}{{D}_{{\varGamma }_{i,i}}^{2}}\right)\right] \right].$$

The first term involves ${\mathbf{G}}_{MF}$ and is simple to manipulate. The last term implies individual terms of Mendelian sampling variances ${D}_{{\Gamma }_{i,i}}$ and it is difficult to compute and derive algebraically in a recognizable form. Instead, we approximate $\frac{\partial lo{g}_{L}\left({\varvec{\Gamma}}\right)}{\partial \gamma }$ by its first term as:

$$\begin{array}{c}\frac{\partial lo{g}_{L}\left({\varvec{\Gamma}}\right)}{\partial \gamma }\approx -\frac{k}{2}\left[Tr\left[{{\varvec{\Gamma}}}^{-1}\frac{\partial{\varvec{\Gamma}}}{\partial \gamma }\left(\mathbf{I}-{{\varvec{\Gamma}}}^{-1}{\mathbf{G}}_{MF}\right)\right]\right]\end{array}.$$

### Derivation of the pseudo-EM algorithm

Consider the approximation above:

$$\begin{array}{l}\frac{\partial lo{g}_{L}\left({\varvec{\Gamma}}\right)}{\partial \gamma }\approx -\frac{k}{2}\left[Tr\left[{{\varvec{\Gamma}}}^{-1}\frac{\partial{\varvec{\Gamma}}}{\partial \gamma }\left(\mathbf{I}-{{\varvec{\Gamma}}}^{-1}{\mathbf{G}}_{MF}\right)\right]\right]\end{array},$$

setting to 0 and factorizing $-\frac{k}{2}$ and introducing ${\gamma }_{ij}$ to indicate which of the points of ${\varvec{\Gamma}}$ we work with gives:

$$Tr\left[{{\varvec{\Gamma}}}^{-1}\frac{\partial{\varvec{\Gamma}}}{\partial {\gamma }_{ij}}\right]=\left[Tr\left[{{\varvec{\Gamma}}}^{-1}\frac{\partial{\varvec{\Gamma}}}{\partial {\gamma }_{ij}}{{\varvec{\Gamma}}}^{-1}{\mathbf{G}}_{MF}\right]\right].$$

Now we focus on $Tr\left[{{\varvec{\Gamma}}}^{-1}\frac{\partial{\varvec{\Gamma}}}{\partial {\gamma }_{ij}}\right]$. In fact, $\frac{\partial{\varvec{\Gamma}}}{\partial {\gamma }_{ij}}$ is a matrix $E\left(i,j\right)$ that contains 1 in the $\left(i,j\right)$ position and 0 otherwise. The *direct* product ${{\varvec{\Gamma}}}^{-1}\odot E\left(i,j\right)$ produces a matrix with 0 s and picking up elements (i,j) of the matrix ${{\varvec{\Gamma}}}^{-1}$. Then we use the fact that:

$$Tr\left(\mathbf{A}\mathbf{B}\right)=\sum \sum {a}_{ij}{b}_{ij}=\sum_{all\; elements}\left(\mathbf{A}\odot \mathbf{B}\right),$$

to observe that in fact $Tr\left[{{\varvec{\Gamma}}}^{-1}\frac{\partial{\varvec{\Gamma}}}{\partial {\gamma }_{ij}}\right]=Tr\left[{{\varvec{\Gamma}}}^{-1}E\left(i,j\right)\right]$ as follows:

for $i=j$ (diagonal elements) then we obtain the diagonal element ${\gamma }^{ij}$ (the element $\left(i,j\right)$ of ${{\varvec{\Gamma}}}^{-1}$, not of ${\varvec{\Gamma}}$);

for $i\ne j$ (off-diagonal elements) then we obtain $2{\gamma }^{ij}$ (because there are two $1$ s then in $E\left(i,j\right)$).

Now we focus on the expression  $Tr\left[{{\varvec{\Gamma}}}^{-1}\frac{\partial \Gamma }{\partial {\gamma }_{ij}}{{\varvec{\Gamma}}}^{-1}{\mathbf{G}}_{MF}\right]=Tr\left[\frac{\partial{\varvec{\Gamma}}}{\partial {\gamma }_{ij}}{{\varvec{\Gamma}}}^{-1}{\mathbf{G}}_{MF}{{\varvec{\Gamma}}}^{-1}\right]$. By the same reasoning, when we consider ${\gamma }_{ij}$ we obtain:

for $i=j$ (diagonal elements) we get the element $\left(i,j\right)$ of ${{\varvec{\Gamma}}}^{-1}{\mathbf{G}}_{MF}{{\varvec{\Gamma}}}^{-1}$, i.e. ${{\varvec{\Gamma}}}^{-1}{\mathbf{G}}_{MF}{{\varvec{\Gamma}}}^{-1}\left[i,j\right]$;

for $i\ne j$ (diagonal elements) then we get *twice* the element $\left(i,j\right)$ of ${{\varvec{\Gamma}}}^{-1}{\mathbf{G}}_{MF}{{\varvec{\Gamma}}}^{-1}$, i.e. $2{{\varvec{\Gamma}}}^{-1}{\mathbf{G}}_{MF}{{\varvec{\Gamma}}}^{-1}\left[i,j\right]$.

Thus, for each $\left(i,j\right)$ combination we have one (for $i=j$ in the position $\left(i,i\right)$) or two (for $i\ne j$, in the positions $\left(i,j\right)$ and $\left(j,i\right)$) scalar equations of the form:

$${\gamma }^{ij}={{\varvec{\Gamma}}}^{-1}{\mathbf{G}}_{MF}{{\varvec{\Gamma}}}^{-1}\left[i,j\right],$$

(note that for $i\ne j$ the factor of 2 cancels out). Putting all these equations together we obtain:

$${{\varvec{\Gamma}}}^{-1}={{\varvec{\Gamma}}}^{-1}{\mathbf{G}}_{MF}{{\varvec{\Gamma}}}^{-1},$$

which after pre- and post-multiplication by ${\varvec{\Gamma}}$ results in:

$${\varvec{\Gamma}}={\mathbf{G}}_{MF}.$$
